# Induced Expression of kir6.2 in A1 Astrocytes Propagates Inflammatory Neurodegeneration via Drp1-dependent Mitochondrial Fission

**DOI:** 10.3389/fphar.2020.618992

**Published:** 2021-01-28

**Authors:** Nanshan Song, Hong Zhu, Rong Xu, Jiaqi Liu, Yinquan Fang, Jing Zhang, Jianhua Ding, Gang Hu, Ming Lu

**Affiliations:** ^1^ Jiangsu Key Laboratory of Neurodegeneration, Department of Pharmacology, Nanjing Medical University, Nanjing, China; ^2^ Department of Pharmacology, Nanjing University of Chinese Medicine, Nanjing, China; ^3^ Neuroprotective Drug Discovery Key Laboratory, Department of Pharmacology, Nanjing Medical University, Nanjing, China

**Keywords:** neuroinflammation, astrocytes, mitochondrial fission, Parkinson’s diseases, kir6.2

## Abstract

Glia-mediated inflammatory processes are crucial in the pathogenesis of Parkinson’s disease (PD). As the most abundant cells of the brain and active participants in neuroinflammatory responses, astrocytes largely propagate inflammatory signals and amplify neuronal loss. Hence, intensive control of astrocytic activation is necessary to prevent neurodegeneration. In this study, we report that the astrocytic kir6.2, as a abnormal response after inflammatory stimuli, promotes the reactivity of A1 neurotoxic astrocytes. Using kir6.2 knockout (KO) mice, we find reversal effects of kir6.2 deficiency on A1-like astrocyte activation and death of dopaminergic neurons in lipopolysaccharide (LPS)-induced mouse models for PD. Further *in vitro* experiments show that aberrant kir6.2 expression induced by inflammatory irritants in astrocytes mediates the dynamin-related protein 1 (Drp1)-dependent excessive mitochondrial fragmentation and results in mitochondrial malfunctions. By deleting kir6.2, astrocytic activation is reduced and astrocytes-derived neuronal injury is prevented. We therefore conclude that astrocytic kir6.2 can potentially elucidate the pathology of PD and promote the development of therapeutic strategies for PD.

## Introduction

Parkinson’s disease (PD) is a common neurodegenerative disease characterized by progressive dopaminergic neurondegeneration and concomitant glial activation in the substantia nigra pars compacta (SNc) (Poewe et al., 2017). Glia-mediated inflammatory processes have long been considered as damage signal amplifiers to propagate the neuronal loss, in which pathogenic and dangerous stimuli provoke pernicious glial reactivity; subsequently, inevitable neuronal injury occurs because of the self-amplifying cycle of glia–neuron crosstalk (Jha et al., 2018; Bernaus et al., 2020). Therefore, excessive propagation of neuroinflammation must be precisely controlled to prevent the death of neurons. Over the last two decades, studies have demonstrated that animal models injected with lipopolysaccharide (LPS) into the SNc can replicate some characteristics of PD. These features include extensive glial activation and selective loss of dopaminergic neurons in the nigrostriatal system, thus it is commonly used for the study of inflammatory mechanisms of PD (Duty and Jenner, 2011; Batista et al., 2019).

Owing to the sensitivity to ATP/ADP ratio, ATP-sensitive potassium (KATP) channels are called metabolic receptors. They primarily function in high energy-demanding tissues and cell types (Tinker et al., 2018). Nevertheless, high energy-consuming tissues such as brain are vulnerable to metabolic dysregulation and chronic inflammation. These two processes promote the progression of neurodegenerative diseases, including PD (Cunnane et al., 2020). Kir6.2, is highly associated with the degeneration of dopaminergic neurons induced by chronic metabolic stress (Liss et al., 2005). We have shown in previous studies that kir6.2 deletion and kir6.2-composing KATP channel inhibition alleviated the neurotoxin-induced PD pathology; such improvement verifies the critical roles of kir6.2-KATP channel in metabolic stress of PD (Zhang et al., 2018b; Zhou et al., 2018). Kir6.2 has also been suggested as a inflammatory mediator, participating in inflammasome regulation in liver injury (Du et al., 2014). As the close link between metabolic dysfucntions and inflammatory responses as well as their critical roles in PD, kir6.2, at the intersection of metablolism sensitivity and inflammation regulation, may be a promising target for the comprehension of the complicated pathogenesis of PD.

Induced by activated microglia in LPS stimulation, neurotoxic A1 astrocytes have recently been reported to result in the death of neurons in neurodegenerative disorders (Liddelow et al., 2017). *In situ* hybridization and immunochemistry experiments revealed that A1 astrocytes compromise a large proportion of astrocytes in the post-mortem tissues from patients with PD, suggesting that this type of astrocytes may be critical for disease initiation and progression (Liddelow et al., 2017; Hinkle et al., 2019). A1 astrocytes show mitochondrial dysfunctions, which can be reversed by treatment with P110, a selective inhibitor of excessive mitochondrial (Joshi et al., 2019). However, the trigger for this unchecked inflammatory phenotype of astrocytes and the internal mechanisms for its being remains undetermined.

In the present study, we investigated the inflammatory mechanisms of neuronal degeneration in PD mice model by injecting LPS into the SNc of the mice. We showed an induced expression of kir6.2 in reactive astrocytes. Our data demonstrated that kir6.2 deletion protected astrocytes from the upregulation of A1-specific markers, as well as astrocytic mitochondrial dysfunctions induced by microglia-conditioned medium (MCM) of LPS treatment. We further found that kir6.2 knockout reduced the mitochondrial translocation of dynamin-related protein 1 (Drp1) and prevented excessive mitochondrial fission in A1 astrocytes. Moreover, inhibition of the A1 astrocyte phenotype by kir6.2 deficiency prevented the death of mesencephalic primary neurons. We conclude that kir6.2, as a critical mediator of the neurotoxic A1-like astrocyte phenotype, is highly involved in the neurological impairments induced by neuroinflammation. Thus, kir6.2 can be a potential target for the development of new strategies for the treatment of PD.

## Materials and Methods

### Animals and LPS-Induced Mouse Model for PD

Kir6.2 knockout mice (kir6.2 KO, kir6.2^−/−^) aged 3 months, with body weights ranging from 24 to 26 g were donated by Professor Miki (Chiba University, Japan). C57BL/6J mice (aged 3 months) were obtained from Comparative Medicine Centre of Yangzhou University. The mice were bred and maintained in the Animal Resource Centre of the Faculty of Medicine, Nanjing Medical University. Mice had free access to food and water in a room with an ambient temperature of 22 ± 2°C and a 12 h:12 h light/dark cycle. All animal experiments were approved by the review committee from Nanjing Medical University and were in compliance with institutional guidelines.

For LPS-induced PD model, mice were anesthetized with pentobarbital sodium (40 mg/kg). The midbrains of the matched WT and kir6.2^−/−^ mice were bilaterally microinjected with LPS (0.5 μg in 1 μl of saline into unilateral brain, 0.2 μl min^−1^) using the following coordinates relative to the bregma: A/P −3.0 mm, R/L ± 1.3 mm and D/V −4.5 mm. The respective controls were injected with equivalent volumes of saline. Seven days later, behavioral analysis were carried out. The animals were then anesthetized and the tissues were harvested for the subsequent bioassay.

### Behavioral Assessment

#### Open Field Test

We conducted open field test to assess the locomotor activity of LPS-induced mouse models for PD. The mice were acclimatized in the behavioral procedure room for 30 min. Spontaneous activity was video-recorded for 5 min in a plastic cage (20 cm × 20 cm × 15 cm) with an outlined center area (10 cm × 10 cm). The mice were individually placed into the open field which was cleaned with 75% (vol/vol) ethanol to minimize olfactory cues in the experimental interval between each animal. The software Top Scan Version 2.0 was used to quantify the overall distance travelled (in cm) and running velocity (in cm/s) as indices of motor activity.

#### Rotarod Test

The rotarod test was used to evaluate mouse limb motor balance and coordination. All the animals were pretrained for three consecutive times at an interval of 20 min before starting the experiment. The experimental rod accelerated from 5 to 25 rpm within 5 min in the adaptation phase. After completing the training trials, the experimental mice were placed in a separate compartment on the accelerating rotarod rod and then tested at 20 rpm within 3 min. The trial ended if the animal fell off the rod or gripped the device without attempting to walk on the rods. Latency to fall was recorded and analyzed.

#### Pole Test

Pole test was performed to evaluate bradykinesia in mice. The mice were placed head upward on a vertical pole (1 cm in diameter and 50 cm in height). The time for mice to turn head downward (T-turn) and the total climbing time taken to reach the base of the pole until the four paws reached on the ground were recorded. The maximum time was 60 s. The mice were trained for three times and results for turning time and climbing down time (in seconds, s) were recorded.

### Primary Cell Cultures and Treatments

Primary astrocyte cultures were performed as described in a previous report (Wei et al., 2020), the brain tissues of the WT and kir6.2^−/−^ neonatal mice aged 1–3 days were stripped of meninges and blood vessels under a microscope. The tissues were then disgested with 0.25% trypsin (Gibco, 27250018) for 2 min and terminated by Dulbecco’s modified Eagle’s medium (DMEM, Gibco, 12100-046) supplemented with 10% fetal bovine serum (FBS, Gibco, 10437028). The cell suspension was filtered with a 40 μm filter (BD falcon, 352340) and centrifuged at 1,000 g for 5 min. Cells were resuspended in DMEM supplemented with 10% FBS and 1% penicillin-streptomycin (Gibco, 15640055) and then plated in culture dishes (Corning, 430167). The culture medium was replaced with fresh medium after 24 h and then refreshed every 3 days. After growing to 90% on the 7th–9th day, the cells were splited into culture plates as needed. Primary astrocytes were treated with miroglia-conditioned media (MCM) mixed with a serum-free DMEM at a ratio of 1:3 for 24 h.

Mesencephalic primary neuron cultures were prepared from the midbrains of embryonic day 16 (E16) C57BL/6J mice. The meninges and blood vessels were removed under the microscope. The separated brain tissue was transferred into fresh high glucose DMEM (Gibco, 12100-046). The tissues were digested with 0.125% trypsin (Gibco, 27250018) at 37°C for 5 min, and terminated with high-glucose DMEM supplemented with 10% FBS (Gibco, 10437028). The cell suspension was filtered with a 40 μm filter (BD falcon, 352340) and centrifuged at 1,000 g for 5 min. The cell precipitate was resuspended in Neurobasal Medium (Gibco, 21103049) supplemented with 2% B27 (Gibco, 17504044), 1% penicillin-streptomycin (Gibco, 15640055) and 0.5 mM glutamine (Gibco, 25030081) and then seeded into cell culture dishes pre-coated with 0.1 mg/ml poly-L-lysine (Sigma, p0296). The medium was renewed every 3.5 days and the cells were treated on Day 7. Mesencephalic primary neurons were treated with astrocyte-conditioned media (ACM) mixed with a neurobasal medium at a ratio of 1:2 for 12 h.

### Immunohistochemical (IHC) Analysis

The brain tissues were dehydrated with 20% sucrose-phosphate buffered saline (PBS) and then 30% sucrose-PBS for three days respectively afte they were fixed in 4% paraformaldehyde. The brains were sliced into sections with a thickness of 25 μm. For immunohistochemical analysis, brain sections were incubated with 3% hydrogen peroxide to quench the endogenous peroxidase activity before blocking with 5% BSA/PBST. After incubation with the primary antibody at 4°C overnight, the HRP-labeled secondary antibody (1:5,000, KPL) was incubated at room temperature for 1 h. The slices were then rinsed with PBS for three times. The slices were finally visualized by the Diaminobenzidine (DAB, Boster, AR1002) reaction for 5 min. Stereo Investigator software was used to visualize and count the number of positive cells under the microscope (Olympus BX51).

The primary antibodies used for IHC staining were as follows: mouse anti-TH antibody (1:1,000, Sigma, T229), mouse anti-GFAP antibody (1:1,000, Millipore, MAB360), rabbit anti-Iba-1 antibody (1:1,000, Wako, 019-19741).

### Nissl Staining

The sections were rinsed with PBS and mounted onto adhesive slides. The slices were treated with Cresyl Violet acetate solution (0.1 g cresyl violet, 1 ml acetic acid in 99 ml H_2_O) for 30 min at room temperature followed by dehydration with a graded series of alcohol and xylene. The brain slices were observed under stereomicroscope (Olympus BX51, Japan).

### Immunofluorescent Analysis

For immunofluorescence of brain slices, the sections were blocked with 5% FBS in PBST (0.3% Triton X-100 in PBS) for 1 h, followed by overnight incubation with primary antibody at 4°C. The sections were then washed with PBS and incubated with Alexa Fluor 488-conjugated goat anti-rabbit (1:1,000, Invitrogen, A11008) or Alexa Fluor 555 goat anti-mouse (1:1,000, Invitrogen, A21422) for 1 h at room temperature. The sections were ultimately rinsed with PBS and then mounted onto adhesive slides. Fluorescently labeled sections were visualized using an Olympus scanning microscope (Olympus BX51, Japan). For immunocytochemical staining, primary cells were rinsed with 0.1 M PBS and fixed with 4% paraformaldehyde for 20 min. Cell cultures on the cell slides were then prepared using the same procedures for the immunofluorescence of the brain slices.

The primary antibodies used for immunofluorescent staining were as follows: rabbit anti-TH antibody (1:1,000, abcam, ab6211), rabbit anti-GFAP antibody (1:1,000, abcam, ab7206), rabbit anti-Iba-1 antibody (1:1,000, Wako, 019-19741), rabbit anti-NeuN (1:100, CST, 24307), mouse anti-kir6.2 antibody (1:200, Santa Cruz, sc-390104), rabbit anti-C3 antibody (1:100, abcam, ab11887), mouse anti-GFAP antibody (1:1,000, Millipore, MAB360), mouse anti-Drp1 (1:200, Santa Cruz, sc-101270) and rabbit anti-TOM20 (1:200, Proteintech, 11802-1-AP).

### Western Blotting Analysis

Mouse brain tissues and cell culture extract lysates were quantified by QuantiPro™ BCA Assay Kit (Sigma, QPBCA). 30 μg proteins were separated by sodium dodecyl sulfate polyacrylamide gel electrophoresis and then electrophoretically transferred to polyvinylidene difluoride (PVDF) membranes (Millipore, IPVH00010). After blocking with 10% nonfat dry milk in Tris-buffered saline (20 mM Tris-HCl, 500 mM NaCl, pH 7.4) with Tween 20 (Aladdin, T104863), the membranes were then probed with the following primary antibodies overnight at 4°C: mouse anti-GFAP antibody (1:1,000, Millipore, MAB360), mouse anti-TH antibody (1:1,000, Sigma, T229), rabbit anti-C3 antibody (1:1,000, abcam, ab11887), mouse anti-kir6.2 antibody (1:1,000, Santa Cruz, sc-390104), mouse anti-Fis1 antibody (1:1,000, Santa Cruz, sc-376447), mouse anti-Drp1 antibody (1:1,000, Santa Cruz, sc-101270), rabbit anti-Drp1 antibody (1:1,000, Proteintech, 12957-1-AP), rabbit anti-COX IV antibody (1:1,000, CST, 4850), rabbit anti-Bcl-2 antibody (1:1,000, Bioworld, BS511), rabbit anti-Bax antibody (1:1,0000, Proteintech, 50599-2-Ig), rat anti-DAT antibody (1:800, Santa Cruz, sc-32258) and mouse anti-β-actin antibody (1:3,000, Sigma, a1978). The membranes were subsequently incubated with a horseradish peroxidase-conjugated goat anti-mouse secondary antibody (1:5,000, Thermo, 31430) or goat anti-rabbit IgG secondary antibody (1:5,000, Thermo, 31460) for 1 h. After being washed, the membranes were scanned and analyzed using an Image Quant LAS 4000 Chemiluminescence Imaging System (GE Healthcare, United States) by chemiluminescence (ECL) western blotting detection reagents Pierce™ ECL (Thermo, 32132).

### RNA Isolation and Quantitative Real Time PCR (RT-PCR)

Total RNA was extracted from midbrain tissue and primary astrocytes using Trizol Reagent (Invitrogen, 15596026) and then reversely transcribed into cDNA using PrimeScript™ RT Master Mix (Takara, RR036A). Real-time PCR was performed in a 10 μl reaction system containing 1,000 μg cDNA, primers, and SYBR (Roche, 04913914001) with the ABI system. GAPDH was used as an internal control gene (Table 1).

**TABLE 1:** The sequences of qPCR primers were as follows.

Gene	Forward Primer	Reverse Primer
C1qa	AAA​GGC​AAT​CCA​GGC​AAT​ATC​A	TGG​TTC​TGG​TAT​GGA​CTC​TCC
C1qb	AAG​ATC​CAG​AAA​CAC​AAG​TCC​CT	CCT​CCT​CAC​CAT​CAA​ATG​TTG​G
il-1a	CGA​AGA​CTA​CAG​TTC​TGC​CAT​T	GAC​GTT​TCA​GAG​GTT​CTC​AGA​G
il-1b	TCA​GGC​AGG​CAG​TAT​CAC​TC	CAT​GAG​TCA​CAG​AGG​ATG​GG
il-6	CCC​CAA​TTT​CCA​ATG​CTC​TCC​T	CAT​AAC​GCA​CTA​GGT​TTG​CCG
Tnf	CCCAC GTCGT AGCAA ACCA	GGCAG AGAGG AGGTT GACTT
il-4	AGA​TGG​ATG​TGC​CAA​ACG​TCC​TCA	AAT​ATG​CGA​AGC​ACC​TTG​GAA​GCC
il-10	ATT​TGA​ATT​CCC​TGG​GTG​AGA​AG	CAG​GGG​AGA​AAT​CGA​TGA​CA
il-33	TGA​GAC​TCC​GTT​CTG​GCC​TC	CTC​TTC​ATG​CTT​GGT​ACC​CGA​T
Arg	CTC​CAA​GCC​AAA​GTC​CTT​AGA​G	AGG​AGC​TGT​CAT​TAG​GGA​CAT​C
H2-T23	GGA​CCG​CGA​ATG​ACA​TAG​C	GCA​CCT​CAG​GGT​GAC​TTC​AT
Serping1	ACA​GCC​CCC​TCT​GAA​TTC​TTT	GGA​TGC​TCT​CCA​AGT​TGC​TC
H2-D1	TCC​GAG​ATT​GTA​AAG​CGT​GAA​GA	ACA​GGG​CAG​TGC​AGG​GAT​AG
Ggta1	GTG​AAC​AGC​ATG​AGG​GGT​TT	GTT​TTG​TTG​CCT​CTG​GGT​GT
Ligp1	GGG​GCA​ATA​GCT​CAT​TGG​TA	ACC​TCG​AAG​ACA​TCC​CCT​TT
Gbp2	GGG​GTC​ACT​GTC​TGA​CCA​CT	GGG​AAA​CCT​GGG​ATG​AGA​TT
Fbln5	CTT​CAG​ATG​CAA​GCA​ACA​A	AGG​CAG​TGT​CAG​AGG​CCT​TA
Ugt1a	CCT​ATG​GGT​CAC​TTG​CCA​CT	AAA​ACC​ATG​TTG​GGC​ATG​AT
Fkbp5	TAT​GCT​TAT​GGC​TCG​GCT​GG	CAG​CCT​TCC​AGG​TGG​ACT​TT
Psmb8	CAG​TCC​TGA​AGA​GGC​CTA​CG	CAC​TTT​CAC​CCA​ACC​GTC​TT
Srgn	GCA​AGG​TTA​TCC​TGC​TCG​GA	TGG​GAG​GGC​CGA​TGT​TAT​TG
Amigo2	GAG​GCG​ACC​ATA​ATG​TCG​TT	GCA​TCC​AAC​AGT​CCG​ATT​CT
C3	CCA​GCT​CCC​CAT​TAG​CTC​TG	GCA​CTT​GCC​TCT​TTA​GGA​AGT​C
gapdh	TGT​AGA​CCA​TGT​AGT​TGA​GGT​CA	AGG​TCG​GTG​TGA​ACG​GAT​TTG

### Cell Viability Assay

The mesencephalic primary neurons were seeded in 96 well plates with a density of 40,000 cells/well. After the treatment, the culture medium was removed, and the suspension of 90 μl fresh culture medium and 10 μl CCK8 solution (Beyotime, c0037) was added into each well. Cell viability was detected by absorbance at 450 nm.

### Hoechst Staining

Cells were stained with Hoechst 33,342 (1 μl diluted in 500 μl PBS) for 10 min and then observed by fluorescent microscopy (Olympus, Tokyo, Japan).

### Detection of Mitochondrial Functions by Fluorescent Dyes

#### MitoSOX Fluorescent Dye

MitoSOX (Invitrogen, M36008) is a fluorescent dye that can be oxidized by mitochondrial superoxide of live cells and exhibit red fluorescence. After treatment, the culture medium of primary astrocytes was sucked off and the cells were stained with 5 μM MitoSOX fluorescent dye at 37°C in the dark for 10 min. The cells were then rinsed twice and then resuspended with cold PBS containing 1% FBS for flow cytometric analysis. Flow data were analyzed with the FCS Express software (Guava Easy Cyte™8, Millipore, United States).

#### JC-1 Fluorescent Dye

JC-1 fluorescent probe (Invitrogen, T3168) was a membrane-permeable dye used to determine mitochondrial membrane potential. Upon membrane polarization, JC-1 was transformed from mitochondrial aggregates with emission of a strong red fluorescence (Ex = 585 nm, Em = 590 nm) to cytoplasm monomers with green fluorescence (Ex = 514 nm, Em = 529 nm). After the treatment, the culture medium of astrocytes was discarded, and the cells were added with fresh JC-1 solution (final concentration is 10 μg/ml) and incubated at 37°C for 30 min. After being rinsed with PBS for three times, the cells were digested and resuspended in cold PBS containing 1% FBS for flow cytometric analysis. For fluorescence photography, cells need to be cultured on the cell slide. After incubation with JC-1 fluorescence dye, the cell slides were washed and fixed with paraformaldehyde. The cells were then stained with Hoechst for 10 min. Images were observed and photos were taken under a fluorescence microscope.

### Mitotracker Green

Mitotracker green (Beyotime, C1048) was used to stain the mitochondria. After the treatment, astrocytic culture medium was discarded. The cells were incubated with fresh Mitotracker green solution at 37°C for 40 min. High magnification images of mitochondrial morphology were captured under 63× manification using CarlZeiss LSM710 Laser scanning confocal microscope.

### Annexin V-FITC/PI (AV/PI) Flow Cytometry

Apoptosis of mesencephalic primary neurons was detected using Annexin V-FITC/propidium iodide (AV/PI) fluorescent dye (Vazyme, A211-01). The mesencephalic primary neurons were inoculated into 12-well plate. After treatment, the cells were digested and collected by centrifugation. After the cells were washed with sterile PBS, 500 μl of Binding Buffer with 5 μl of AV and 5 μl PI dye were added to each sample to incubate for 5 min. Flow cytometry was conductd for analysis.

### ATP Assay

ATP contents in the primary astrocytes of the WT and kir6.2^−/−^ mice were detected using an Enhanced ATP Assay Kit (Beyotime, S0027) according to the manufacturer’s protocol. Cells were lysed in an ATP lysis buffer and the amount of protein in each sample was homogenized with a lysis buffer after protein quantification. The ATP contents were determined using a luminometer.

### Coimmunoprecipitation (CO-IP) Assay

Proteins from primary astrocytes were lysed in the cell lysis buffer after experimental treatments. Lysates were incubated overnight with the anti-Drp1 antibody, anti-Fis1 antibody, or mouse IgG (CST, 3420) or rabbit IgG (CST, 3423), followed by incubation with protein A/G-agarose beads (Santa Cruz, sc-2003) for another period of 4 h in 4°C. After centrifugation at 500 g for 3 min, the pellet was washed with pre-cooled PBS, and the beads were boiled in loading buffer for 5 min. Then the supernatants were collected and subjected to western blot analysis for Drp1 and Fis1.

### Mitochondrial Protein Extraction

The mitochondria of primary astrocytes were extrated using the Cell Mitochondria Isolation Kit (Beyotime, C3610). Primary astrocytes were digested with trypsin and then centrifuged to collect the pellets. After ice-incubation with 1 ml mitochondrial isolating reagents containing PMSF for 10–15 min, cell suspension was homogenized. The homogenates were centrifuged at 600 g for 10 min at 4°C. The supernatants were then proceeding to centrifuge at 11,000 g for 10 min at 4°C. After centrifugation, the sediments were the mitochondrial part and the supernatants were cytoplasmic part. We centrifuged the supernatants containing the cytoplasmic part at 12,000 g for 10 min at 4°C. The supernatants obtained, which were cytoplasmic proteins without mitochondria, were then collected. Isolated mitochondria were lysed with 150 μl of the mitochondrial lysis buffer (containing PMSF) on ice for 30 min and then the samples were centrifuged at 16,000 g for 15 min at 4°C, the supernatants obatined were the mitochondrial proteins.

### Statistical Analysis

All data were represented as mean ± SEM. of at least three independent experiments. Statistical analysis were performed using GraphPad Prism 7.0. Student’s unpaired two-tailed *t*-test, one-way ANOVA or two-way ANOVA was conducted according to test requirements. Difference was considered significant at *p* < 0.05. The number of replicates and repeats of individual experiments and statistical tests were indicated in the legends.

## Results

### LPS Induces Dopaminergic Neurons Loss and Glial Activation in the SNc of the Mice

**FIGURE 1:**
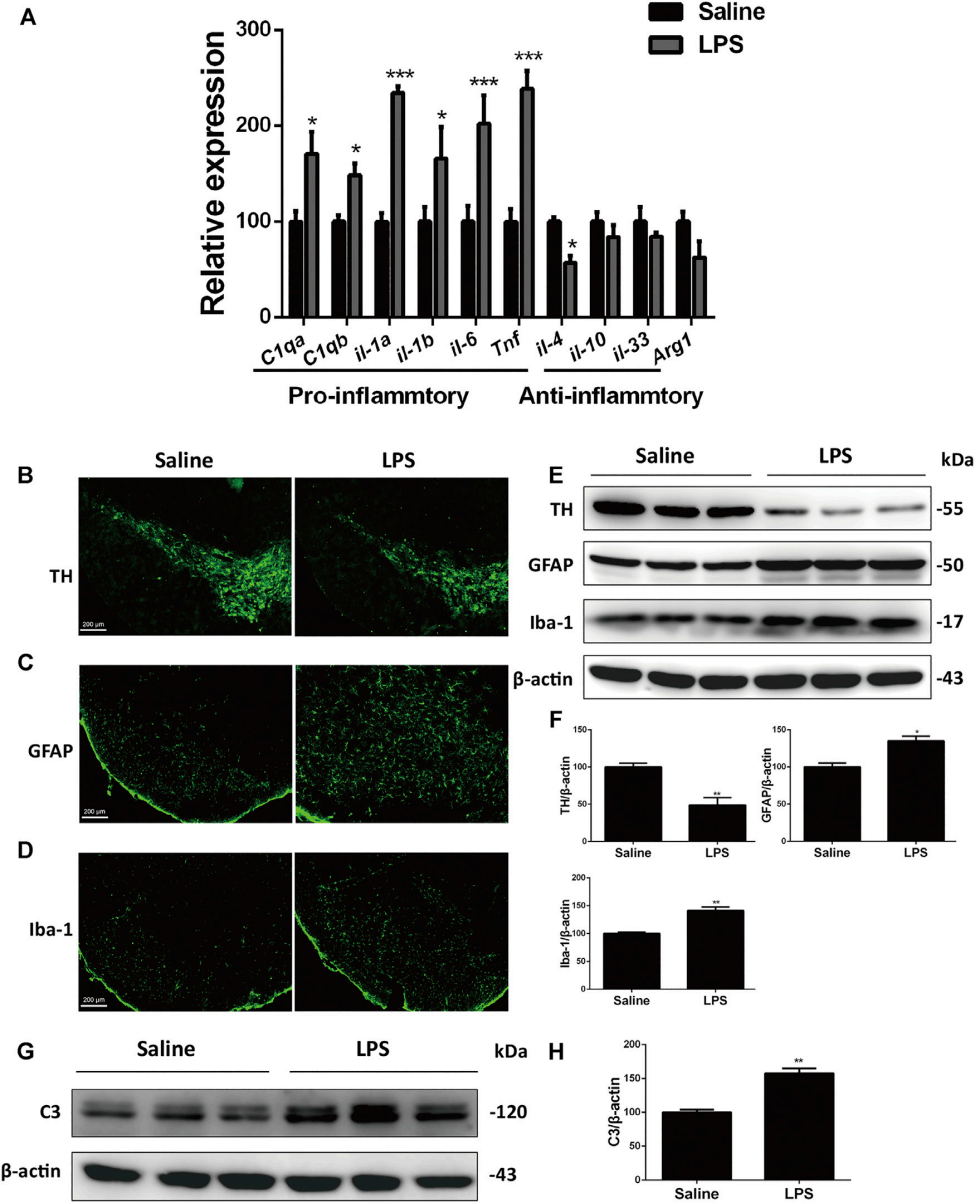
LPS induces dopaminergic neurons loss and glial activation in the SNc of mice. **(A)** WT mice were made LPS-induced PD models by bilaterally microinjection of 0.5 μg LPS in 1 μl saline into the unilateral SNc. mRNA levels of pro-inflammatory and anti-inflammatory genes were analyzed by RT-PCR. **(B)** Immunofluorescent staining of TH^+^ neurons in the SNc. **(C)** Immunofluorescent staining of GFAP in the SNc. **(D)** Immunofluorescent staining of Iba-1 in the SNc. **(E)** Protein levels of TH, GFAP and Iba-1 in brain lysates were analyzed by immunoblot analysis. **(F)** Densitometric analysis of TH, GFAP and Iba-1. **(G)** Expression of C3 in brain lysates was analyzed by immunoblot analysis. **(H)** Densitometric analysis of C3. Data are analyzed by unpaired Student’s test. ^*^ *p* < 0.05, **p < 0.01, and ***p < 0.001 *vs.* the saline group. Values are presented as means ± SEM from three independent experiments.

Neuroinflammation is a predominant feature of the aging brain and most neurodegenerative diseases including PD (Yang and Zhou, 2019; Kam et al., 2020). To study the inflammatory mechanisms of PD, we used the stereotaxic injection of LPS into the SNc, a well-characterized PD inflammatory model, to figure out the link between neuroinflammation and neurodegeneration (Batista et al., 2019). After LPS stimulation, the expression of pro-inflammatory genes in LPS-stimulated mice increased significantly relative to the saline-treated mice, whereas the expression of anti-inflammatory genes decreased or remained unaltered (Figure 1A). This result implied that LPS induced injurious inflammatory responses. In immunofluorescent analysis, we observed obvious loss of dopaminergic neurons, as indicated by decreased number of tyrosine kinase (TH)-positive cells (Figure 1B). LPS also increased activation of astrocytes and microglia in inmmunofluorescent stainings of glial fibrillary acidic protein (GFAP) and ionized calcium-binding adapter molecule 1 (Iba-1), respectively (Figures 1C,D). Immunoblotting analysis of these specific markers consistently showed that LPS induced the decreased expression of TH and increased levels of GFAP and Iba-1 (Figures 1E,F).

Previous studies have verified that LPS-induced neuroinflammation promotes formation of the neurotoxic reactive astrocytes, termed the A1 astrocytes, by activated microglia (Liddelow et al., 2017). In the current sudy, we found that the protein levels of complement component 3 (C3), the most characteristic marker of A1 astrocytes, were upregulated significantly in the SNc of the LPS mouse models compared with that of the saline group mice (Figures 1G,H). These results suggest that LPS induces loss of dopaminergic neurons and glial activation in the SNc.

### Kir6.2 Is Inducibly Expressed in the Reactive Astrocytes of the LPS-Stimulated Mice

**FIGURE 2:**
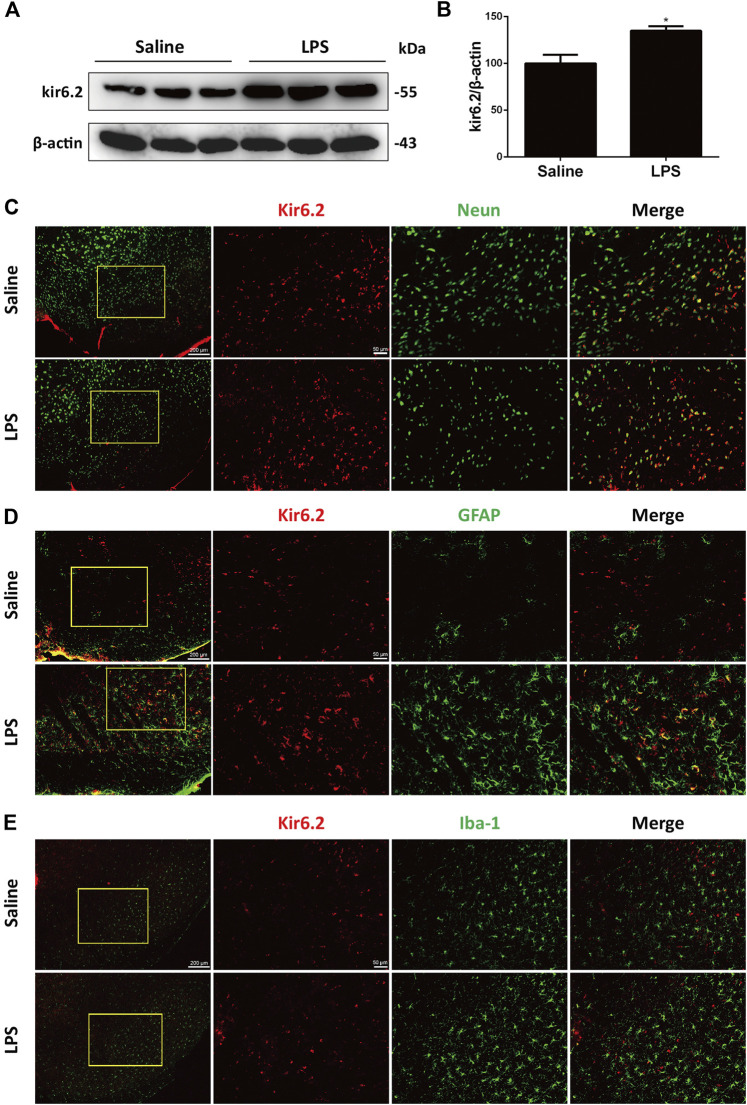
Kir6.2 is inducibly expressed in the reactive astrocytes of LPS-stimulated mice. **(A)** Expression of kir6.2 in saline and LPS-injected brain was measured by western blotting. **(B)** Densitometric analysis of kir6.2. **(C)** Representative immunofluorescent stainings of NeuN (green) and kir6.2 (red) in brain slices of saline and LPS mice were shown. **(D)** Representative immunofluorescent stainings of GFAP (green) and kir6.2 (red) in the brain slices of saline and LPS mice were shown. **(E)** Representative immunofluorescent stainings of Iba-1 (green) and kir6.2 (red) in the brain slices of saline and LPS mice were shown. Data are analyzed by unpaired Student’s test. ^*^ *p* < 0.05 *vs.* the saline group. Values are presented as means ± SEM from three independent experiments.

The kir6.2-KATP channel actively regulates the electrical activity of the dopaminergic neurons and is significantly involved in sustained metabolic stress-induced neurodegeneration (Liss et al., 2005). We previously demonstrated that kir6.2 participated in peripheral inflammatory responses (Du et al., 2014). In this context, we explored the possible roles of kir6.2 in central inflammatory regulation in PD. As shown in Figures 2A,B, LPS significantly upregulated kir6.2 protein levels in the SNc. We next asked whether an increase in kir6.2 was a response of neurons to an inflammatory stimulus. Thus, we measured the kir6.2 expressions in neurons by dual immunofluorescence of kir6.2 and NeuN, the marker of neurons. However, kir6.2 that is not co-labeled with NeuN existed in the LPS group. As glia being the principal effectors of neuroinflammation and most numerous cell types in the brain (Domingues et al., 2020), we therefore speculated that kir6.2 upregulation mainly occurred in activated microglia or reactive astrocytes in the LPS-induced mouse models for PD. Although expression of kir6.2 in glial cells used to be inconclusive, recent studies have demonstrated aberrant expression of kir6.2 in astrocytes under certain pathological conditions (Griffith et al., 2016; Castro et al., 2019). We were promoted to assess the possible localization of kir6.2 in activated microglia or/and astrocytes. Immunofluorescent studies showed that increased kir6.2 was seen in reactive astrocytes, rather than microglia, of the LPS-PD mice brain (Figures 2C,D). Taken together, LPS-induced increase of kir6.2 is an aberrant expression in reactive astrocytes.

### Deficiency of kir6.2 Prevents Dopaminergic Neurons Loss and Behavioral Deficits in LPS-Induced Mouse Model for PD

**FIGURE 3:**
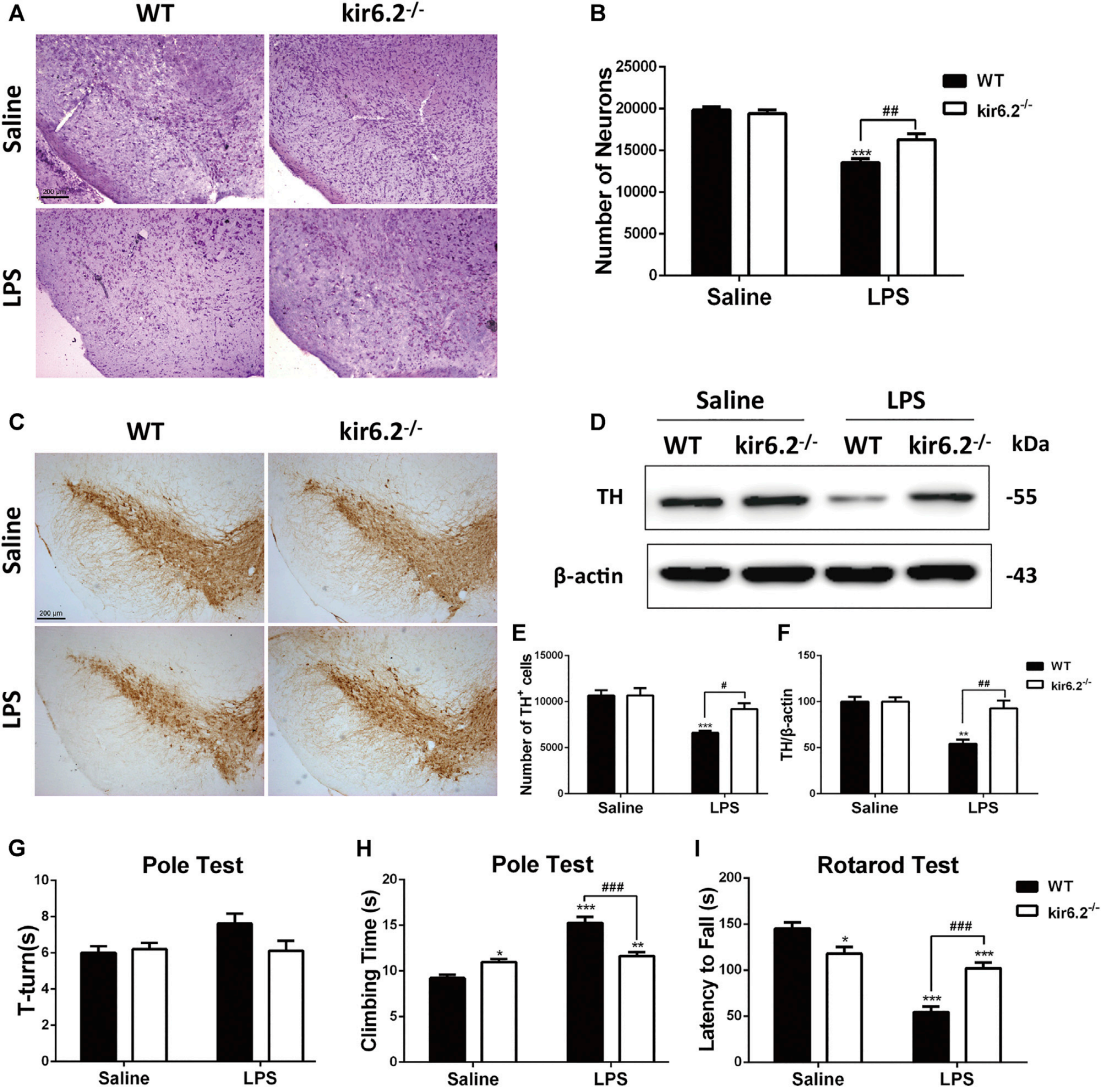
Kir6.2 deficiency prevents dopaminergic neurons loss and behavioral deficits in LPS-induced mouse models for PD. **(A)** Microphotographs of Cresyl violet-positive cells in the SNc. **(B)**Stereological counts of Cresyl violet-positive cells in the SNc. **(C)** Representative IHC staining of TH-positive neurons in midbrain sections. **(D)** Protein levels of TH in brain lysates were analyzed by immunoblotting analysis. **(E)** Stereological counts of TH-positive neurons in the SNc. **(F)** Densitometric analysis of TH. **(G)** Turning time (T-turn) of the mice in the pole test. **(H)** Total climbing time of the mice in the pole test. **(I)** Latency to fall of the mice in the rotarod test. Data were analyzed by two-way ANOVA. *p < 0.05, **p < 0.01, and ***p < 0.001 *vs.* the saline group of the same genotype. ^#^ *p* < 0.05, ^##^ *p* < 0.01 and ^###^ *p* < 0.01 *vs.* the LPS group of the WT mice. *n* = 6 mice per group for IHC and Nissl staining. *n* = 3 for western blotting. *n* = 12 mice for behavioral tests. Values are presented as means ± SEM.

We previously reported that kir6.2 deficiency protected against dopaminergic neurodegeneration in the chronic 1-methyl-4-phenyl-1,2,3,6-tetrahydropyridine (MPTP) mouse model (Zhang et al., 2018b; Zhou et al., 2018), implying that the kir6.2-KATP channel is a potential pathological target for metabolic stress in PD. The regulatory effects of kir6.2 in peripheral inflammation have been identified, but its involvement in neuroinflammatory processes of PD remains undetermined. As we showed the ectopic expression of kir6.2 in reactive astrocytes, we inferred that these changes might subsequently contribute to the inflammatory phenotypes of PD. We established LPS-induced mouse model for PD using kir6.2 knockout mice and their wide-type (WT) counterparts and evaluated the effect of kir6.2 deletion on DA neuron impairment induced by LPS. Stereological counts of SNc total neurons defined by Nissl staining showed no difference in the number of total neurons between both genotypic mice under saline treatment. LPS treatment decreased the Nissl-positive cells by 32% in the SNc of the WT mice, but decreased the Nissl-positive cells by 19% in the kir6.2 KO mice (Figures 3A,B). The reversal effects of kir6.2 deficiency on dopaminergic neuron damage were further confirmed by TH immunostaining and immunoblotting. As shown in Figures 3C,E, LPS treatment decreased the TH-positive cells by 38% in SNc of WT mice, vs. by 14% in SNc of kir6.2-deficient mice. Kir6.2 deletion reversed the LPS-induced decreased TH protein levels in the SNc (Figures 3D,F). We also examined the performance of the kir6.2^−/−^ mice in behavioral coordination and locomotor activity after LPS stimulation. The data from the total climbing time of the pole test and latency to fall in the rotarod test indicated that the kir6.2 KO mice improved the LPS-induced impairment of motor performance ((Figures 3H,I), however the turning time of mice in the pole test did not indicate any significant change between groups (Figure 3G). Consistent with a previous study (Deacon et al., 2006), we found decreased locomotor activities in the kir6.2-deficient mice compared with WT mice in the basal state (Figures 3H,I). This reduction is predicted to be related to the physiological functions of kir6.2 in behavioral control (Schiemann et al., 2012). Together, these results indicated that kir6.2^−/−^ mice are more resistant to LPS-induced neurodegeneration than their control littermates.

### Kir6.2 Deletion Attenuated A1-like Astrocytes Reactivity in LPS-Induced Mouse Models for PD

**FIGURE 4:**
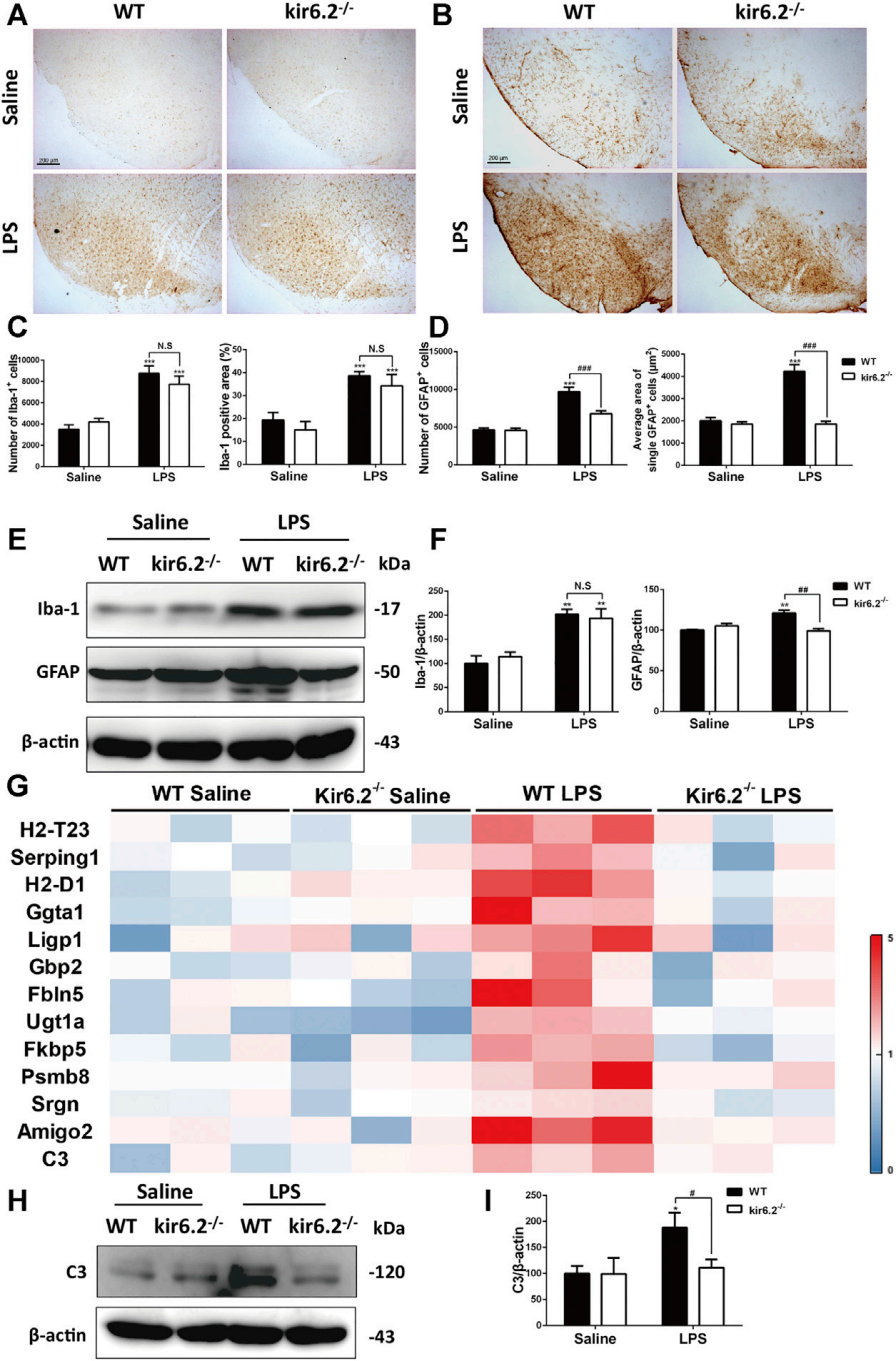
Kir6.2 deletion attenuates A1-like astrocytes reactivity in LPS-induced mouse models for PD. **(A)** Representative IHC staining of Iba-1 in midbrain sections. **(B)** Representative IHC staining of GFAP in midbrain sections. **(C)** Counts of Iba-1^+^ cells and percentage of Iba-1 positive areas in midbrain sections. **(D)** Counts of GFAP^+^ cells and average area of single GFAP^+^ cells in midbrain sections. **(E)** The protein levels of GFAP and Iba-1 in the midbrain tissues were examined by western blotting. **(F)** Densitometric analysis of Iba-1 and GFAP. **(G)** Heat map comparing the mean expression of A1-specific transcripts in mesencephalic RNA samples. **(H)** Expression of C3 in the midbrain were detected by western blotting. **(I)** Densitometric analysis of C3. *p < 0.05, **p < 0.01, and ***p < 0.001 *vs.* the saline group of the same genotype. ^#^ *p* < 0.05, ^##^ *p* < 0.01, and ^###^ *p* < 0.01 *vs.* the LPS group of the WT mice. *n* = 6 mice per group for IHC analysis. *n* = 3 for western blotting and RT-PCR. Values are presented as means ± SEM.

We next detected the activation of microglia and astrocytes by Iba-1 and GFAP immunostaining. As shown in Figures 4A–D, LPS treatment activated both microglia and astrocytes in the SNc of the WT mice, as indicated by increases in the number and amplified body areas of both cells. Kir6.2 knockout showed no effect on LPS-induced microglial activation, but inhibited the astrocytic activation. Additionally, protein levels of Iba-1 and GFAP in the SNc demonstrated that kir6.2 deletion cancelled LPS-induced upregulation of GFAP, but not of Iba-1 (Figures 4E,F). These results indicated the differential regulation of kir6.2 in microglial and astrocytic reactivity. As A1 astrocytes were noted in mice models for PD (Liddelow et al., 2017; Yun et al., 2018), we further detected the specific markers of A1 astrocyte in the LPS-induced PD model of the WT and kir6.2-deficient mice by RT-PCR. The mRNA levels of A1 markers were presented as a heatmap in Figure 4G. The result revealed that LPS treatment absolutely resulted in the formation of A1 astrocytes; meanwhile, kir6.2 deletion recovered the increases in mRNA level of A1 markers in the brain of LPS-injected mice. We also detected protein levels of C3 and found that kir6.2 knockout prevented C3 upregulation induced by LPS stimulation (Figures 4H,I). Together, these findings indicated that kir6.2 deficiency prevents A1-like astrocytes reactivity induced by LPS.

### Kir6.2-Deficient Astrocytes Are Resistant to the Neurotoxic A1-Like Phenotype *In Vitro*

**FIGURE 5:**
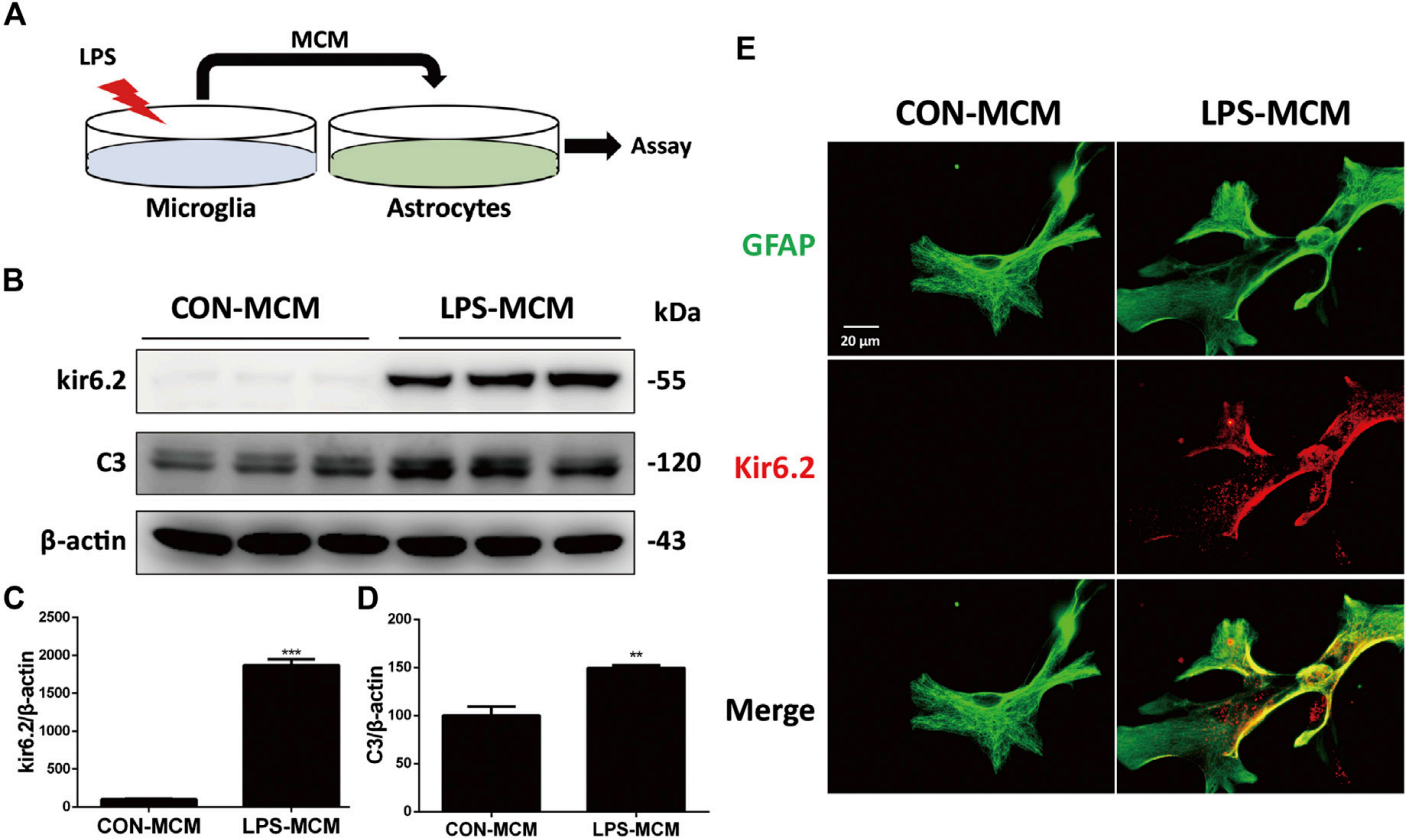
LPS-MCM triggered the aberrant expression of kir6.2 in primary astrocytes. **(A)** Protocol of treatment for **(B–E**). Primary microglia were stimulated with 100 ng/ml LPS for 24 h to collect the MCM. Primary astrocytes were cultured with the MCM diluted at a ratio of 1:3 for 24 h to conduct bioassay. **(B)** Protein levels of kir6.2 and C3 were detected by western blotting. **(C)** Densitometric analysis of kir6.2 and C3. Data are analyzed by unpaired Student’s test. **p < 0.01 and ***p < 0.001 *vs.* CON group. **(D)** Immunofluorescent stainings of GFAP (green) and kir6.2 (red) in primary astrocytes.

We cultured astrocytes and exposed them to the microglia-conditioned medium (MCM) of LPS-treated WT microglia *in vitro* (Figure 5A). Compared with cells exposed to the MCM of untreated microglia, those exposed to the MCM of LPS-treated microglia could sufficiently induce C3 upregulation (Figures 5B,C) and the aberrant expression of kir6.2 in primary astrocytes (Figures 5B,D). Immunofluorescent staining of kir6.2 and GFAP in astrocytes consistently confirmed the existence of kir6.2 in primary astrocytes after LPS-MCM stimulation (Figure 5E).

**FIGURE 6:**
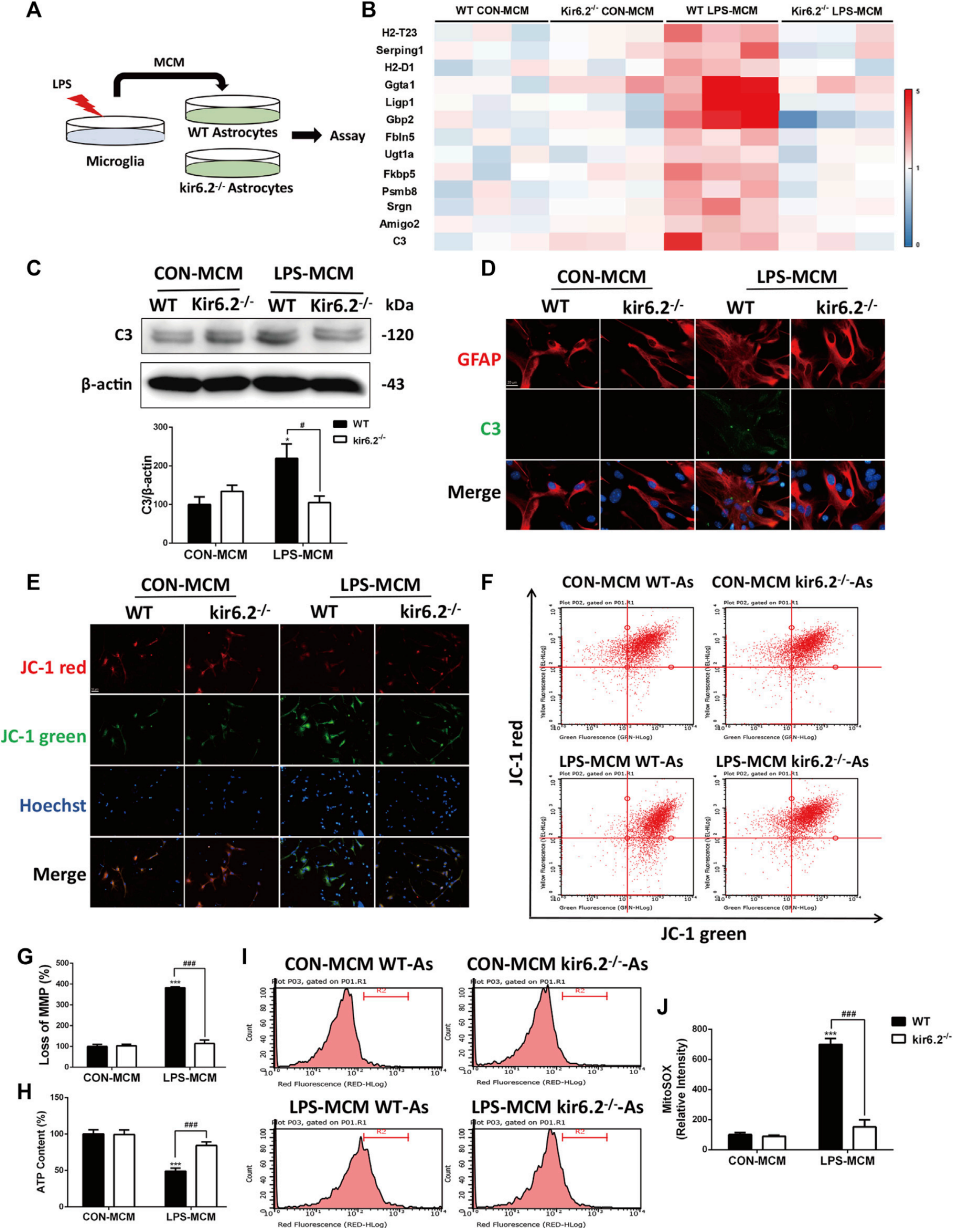
Kir6.2-deficient astrocytes are resistant to neurotoxic A1-like phenotype *in vitro*. **(A)** Protocol of treatment for **(B–J)**. Primary microglia from WT mice were stimulated with 100 ng/mL LPS for 24 h to collect the MCM. For primary astrocytes cultures, the MCM was diluted at a ratio of 1:3 to incubate the primary astrocytes from WT and kir6.2^−/−^ mice for 24 h. **(B)** Heat map comparing the mean expression of A1-specific transcripts in astrocytic RNA samples by RT-PCR. **(C)** Expression of C3 in primary astrocytes detected by Western blotting and its densitometric analysis. **(D)** Immunofluorescent stainings of C3 (green) and GFAP (red) in primary astrocytes. **(E)** Representative images of JC-1 stain in astrocytes were observed by confocal microscopy. Hoechst stains nucleus (blue). **(F)** Flow cytometric analysis of astrocytes stained with JC-1 fluorescent probe. **(G)** Quantification of MMP loss in JC-1 staining measured by flow cytometry. **(H)** ATP contents of astrocytes were analyzed. **(I)** Astrocytes were stained with MitoSOX fluorescent probe and analyzed by flow cytometry. **(J)** Quantification of the mitochondrial ROS by MitoSOX staining. Data were analyzed using two-way ANOVA. *p < 0.05 and ***p < 0.001 vs. corresponding CON-MCM group. ^#^ *p* < 0.05 and ^###^ *p* < 0.001 vs. WT LPS-MCM group. Values are presented as means ± SEM from three independent experiments.

To examine the effects of kir6.2 deficiency on the activation of A1 neurotoxic astrocytes, astrocytes from both the WT and kir6.2^−/−^ mice were cultured and exposed to the MCM of the LPS-treated WT microglia (Figure 6A). With regard to the RT-PCR analysis of A1 markers, we found that kir6.2 deletion prevented the increased mRNA levels of A1 markers induced by the LPS-treated MCM (Figure 6B). Meanwhile, the C3 protein levels were significantly reduced in the kir6.2-deficient astrocytes by immunoblotting analysis (Figure 6C), as well as immunofluorescent double-staining of C3 and GFAP (Figure 6D), suggesting the inhibitory role of kir6.2 deletion in A1 astrocyte formation. A1 pro-inflammatory state of astrocytes are demonstrated to show abnormal mitochondrial functions, as evidenced by loss of normal mitochondrial membrane polarization (MMP), induced ATP levels and increased mitochondrial ROS production (Joshi et al., 2019). Thus, we subsequently detected mitochondrial dysregulations between the WT and kir6.2^−/−^ A1 astrocytes. JC-1 assay system is used to detect MMP, in which tranformation of strong red fluorescence to green fluorescence indicates MMP loss. We observed an drastic MMP disruption in the WT astrocytes treated with LPS-MCM compared with control group, which was corrected by kir6.2 deletion (Figures 6E–G). Moreover, kir6.2 deletion recovered the decreased ATP levels by using the ATP assay kit (Figure 6H) and increased mitochondrial ROS production as detected with MitoSOX fluorescent probe (Figures 6I,J) in astrocytes stimulated with LPS-MCM. Together, our data show that kir6.2 knockout protected astrocytes from the A1 phenotype and mitochondrial dysfunctions.

### Astrocytic kir6.2 Promotes Drp1-dependent Mitochondrial Fission *In Vitro*

**FIGURE 7:**
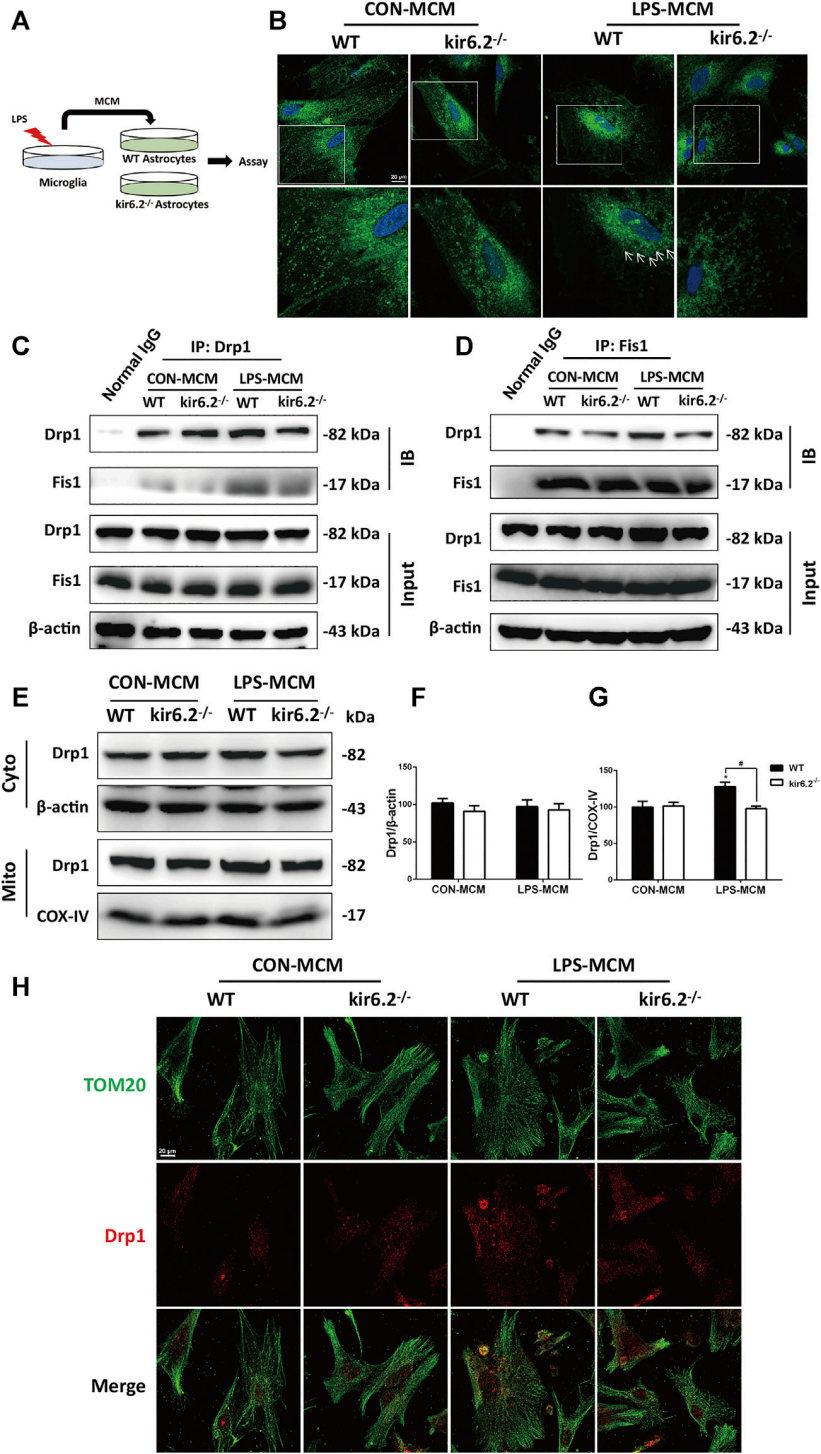
Astrocytic kir6.2 promotes Drp1-dependent mitochondrial fission *in vitro*. **(A)** Protocol of treatment for **(B–J)**. Primary microglia from WT mice were stimulated with 100 ng/ml LPS for 24 h to collect the MCM. For primary astrocytes cultures, the MCM was diluted at a ratio of 1:3 to incubate the primary astrocytes from WT and kir6.2^−/−^ mice for 24 h. **(B)** Representative images of Mitotracker Green in primary astrocytes under CarlZeiss LSM710 Laser scanning confocal microscope. **(C)** Immunoblotting analysis of Fis1 proteins in cell lysates immunoprecipitated with Drp1 antibody. **(D)** Immunoblotting analysis of Drp1 proteins in cell lysates immunoprecipitated with Fis1 antibody. **(E)** Protein levels of Drp1 in cytoplasmic and mitochondrial parts were detected by immunoblots. **(F)** Protein levels of Drp1 in cytoplasmic and mitochondrial parts were detected by immunoblots. **(G)** Densitometric analysis of Drp1 in cytoplasmic and mitochondrial parts. **(H)** Immunofluorescent stainings of TOM20 (green) and Drp1 (red) in primary astrocytes. Data are analyzed using two-way ANOVA. ^*^ *p* < 0.05 *vs.* corresponding CON group. ^#^ *p* < 0.05 *vs.* WT A1 group. Values are means ± SEM from three independent experiments.

A previous study has confirmed that dynamin-related protein 1 (Drp1)-mitochondrial fission 1 (Fis1)-mediated mitochondrial fission is involved in the A1 reactive phenotype of astrocytes (Joshi et al., 2019). Indeed, mitochondrial fragmentation as a consequence of excessive Drp1-induced mitochondrial fission is a prototypical feature of neurodegenerative diseases including PD. To investigate the molecular mechanisms underlying the insusceptibility of kir6.2-deficient astrocytes to the A1 reactive state, we transferred the MCM to the WT and kir6.2^−/−^ primary astrocytes (Figure 7A). We explored the role of Drp1-Fis1-dependent mitochondrial fragmentation process in astrocytic kir6.2-mediated pro-inflammatory astrogliosis. Mitotracker green, a mitochondrial fluorescent probe, was used to examine mitochondrial morphology in primary atrocytes. As exhibited in Figure 7B, LPS-MCM triggered tubular or dotted mitochondrial pattern denoted by white arrows in the WT astrocytes, while kir6.2-deficient A1 astrocytes exhibited normal circular structure, like the healthy mitochondria in control group of the two genotypes. The fission-promoting activity of Drp1 is controlled by the anchorage of Drp1 to Fis1, the receptor in the mitochondrial membrane, to perform mitochondrial fission (Cho et al., 2013); thus we assessed the translocation of Drp1 to the mitochondrial part. Co-IP assay demonstrated that kir6.2 deficiency decreased the interaction between DRP1 and Fis1 in A1 the astrocytes (Figures 7C,D). We separately detected the Drp1 levels of the mitochondrial and cytoplasmic parts. Immunoblotting analysis revealed that mitochondrial Drp1 was significantly upregulated after LPS-MCM stimulation, whereas kir6.2 deletion recovered the mitochondrial Drp1 levels, and the cytoplasmic Drp1 was not changed (Figures 7E–G). We also observed the mislocalization of Drp1 by immunofluorescently double-staining Tom20, the mitochondrial marker, and Drp1. The results in Figure 7H showed that kir6.2 deficiency decreased translocation of DRP1 to the mitochondria induced by LPS-MCM. Taken together, these findings suggested that kir6.2 deletion prevented mitochondrial translocation of Drp1 and saved astrocytes from excessive mitochondrial division.

### Kir6.2-Deficient A1 Astrocytes Fail to Induce Mesencephalic Neuron Injury *In Vitro*

**FIGURE 8:**
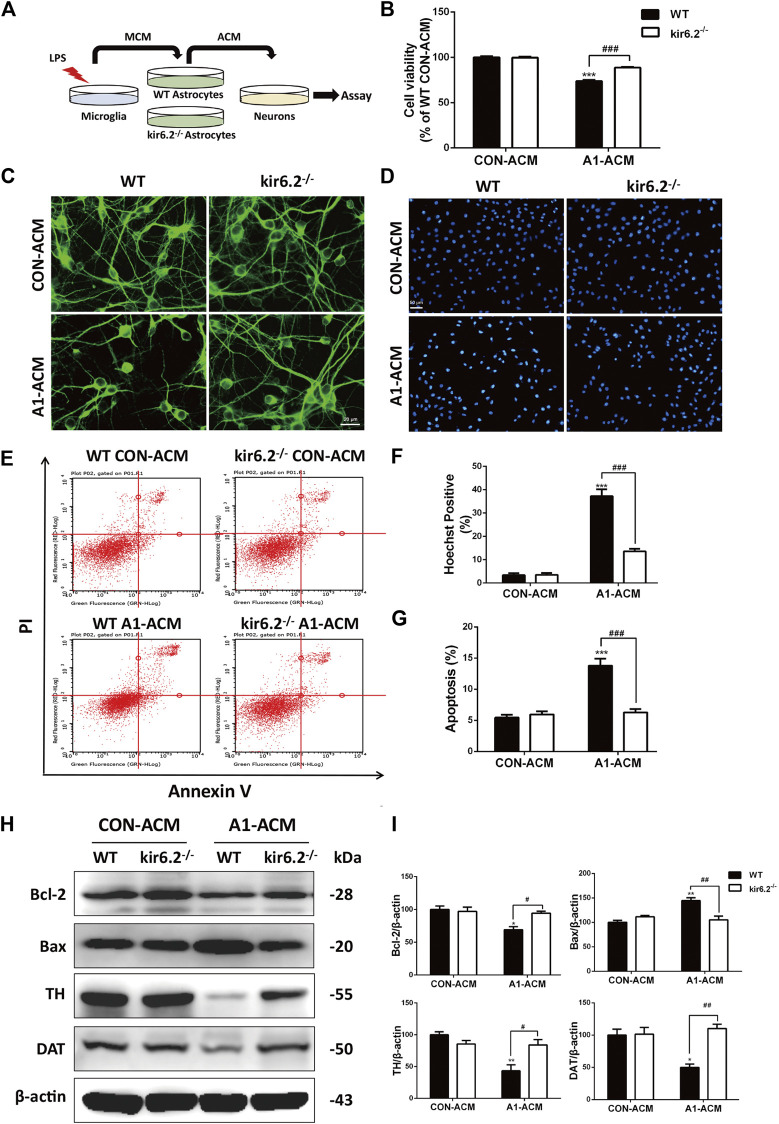
Kir6.2-deficient A1 astrocytes fail to induce injury of mesencephalic neurons *in vitro*. **(A)** Protocol of treatment for **(B–I)**. The mesencephalic primary neurons were incubated with the ACM mixed with neurobasal medium at a ratio of 1:2 for 12 h to conduct the bioassay. **(B)** Cell viability of mesencephalic primary neurons was assayed using CCK8 kit. **(C)** Representative immunofluorescent stainings of MAP2 in primary neurons. **(D)** Representative images of Hoechst-stained nuclei in primary neurons. **(E)** Flow cytometric analysis of primary neurons stained with AV/PI kit. **(F)** Quantification of Hoechst-positive cells was analyzed. **(G)** Quantification of dead cells in the flow cytometric analysis of AV/PI was analyzed. **(H)** Bcl-2, Bax, TH and DAT in the cell extracts of primary neurons were analyzed by immunoblotting analysis. **(I)** Densitometric analysis of Bcl-2, Bax, TH and DAT. Data are analyzed using two-way ANOVA. *p < 0.05, **p < 0.01, and ***p < 0.01 *vs.* corresponding CON-ACM group. ^#^ *p* < 0.05, ^##^ *p* < 0.001, and ^###^ *p* < 0.001 *vs.* WT A1-ACM group. Values are means ± SEM from three independent experiments.

Former studies have suggested that A1 astrocytes contribute to the death of neurons (Liddelow et al., 2017). Therefore, we assessed the neurotoxic effects of the kir6.2-deficient A1 astrocytes on neurons by transferring the conditioned media of microglia-activated WT and kir6.2^−/−^ astrocytes to incubate the mesencephalic primary neurons (Figure 8A). As shown in Figure 8B, A1 astrocyte-conditioned media (ACM) evoked significant decrease in primary neuron viability by CCK8 assay, whereas kir6.2-deficient A1-ACM caused no injury to the neurons. Morphological analysis in MAP2 immunofluorescent staining showed markedly decreased neuronal processes after treatment with WT A1-ACM, and this decrease was not observed in the kir6.2^−/−^ A1-ACM treatment group (Figure 8C). In addition, Hoechst-positive cell numbers (Figures 8D,F) and the AV/PI-positive percentage of total neurons (Figures 8E,G) increased in the group treated with WT A1-ACM relative to that in the group treated with WT CON-ACM. Moreover, apoptosis regulatory proteins including Bcl-2 and Bax changed significantly (Figures 8H,I), indicating the neurotoxic effects of the A1 astrocytes. However, all A1-derived pro-apoptotic effects on primary neurons were blunted when the kir6.2 protein was knocked out from the astrocytes. Protein levels of TH and DAT, the specific markers of dopaminergic neuron, were also detected to validate the injury of neurons. We found A1-MCM from kir6.2-deficient astrocytes failed to decrease the expressions of TH and DAT (Figures 8H,I). Together, kir6.2-deficient A1 astrocytes showed lower toxicity to the mesencephalic neurons.

## Discussion

**FIGURE 9:**
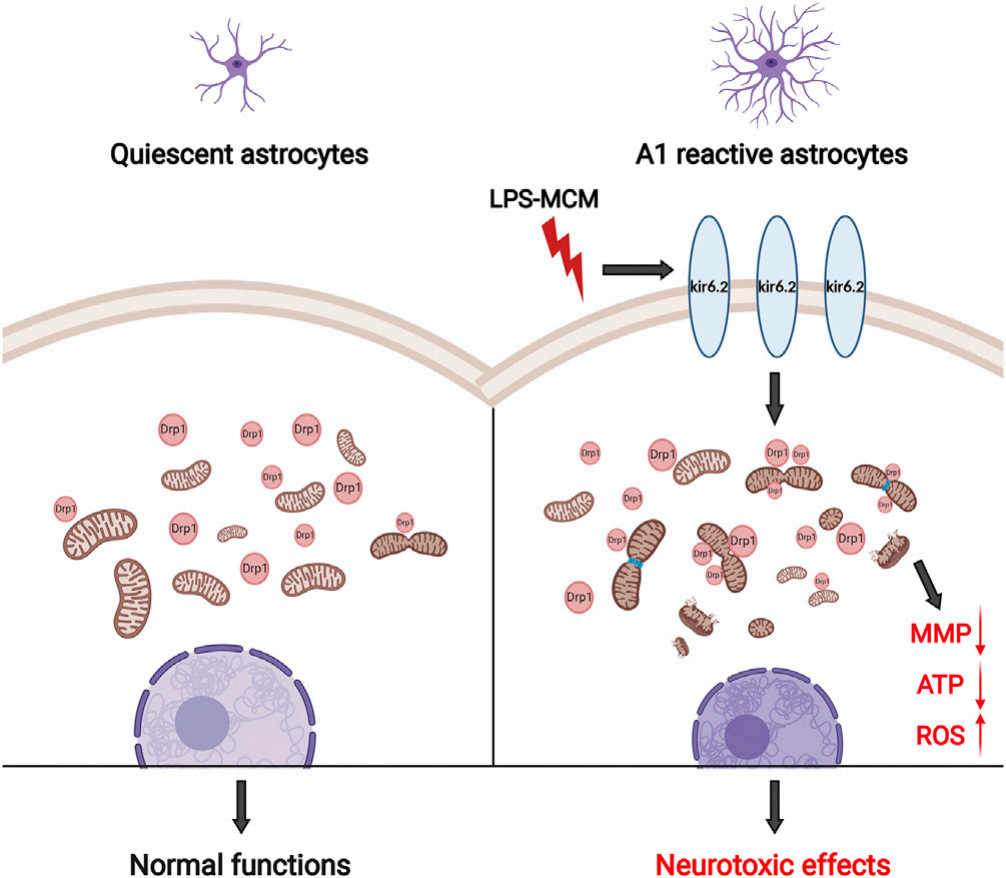
Schematic graph of astrocytic kir6.2 in promoting neurotoxic A1 astrocyte formation. Abnormal expression of kir6.2 in activated astrocytes induced by inflammatory stimuli orchestrates the formation of neurotoxic A1 astrocyte phenotype by promoting Drp1-mediated excessive mitochondrial fission and subsequent mitochondrial malfunctions, including MMP loss, energy supply shortage and oxidative stress.

The present study illustrates a indispensable role of kir6.2 in the inflammatory pathogenesis of PD. Our results demonstrate that LPS induces an increase in kir6.2 expression, which is mainly observed in the reactive astrocytes, rather than the neurons or microglia. We suggest that the abnormal kir6.2 expression in activated astrocytes orchestrates the formation of the neurotoxic A1 astrocytes. In addition, suppression of the pathology-associated kir6.2 by deleting the kir6.2 protein prevents the excessive astrocyte reactivity and the notable loss of dopaminergic neurons in the inflammatory mouse models for PD. Further, the *in vitro* study validates the inhibitory role of astrocytic kir6.2 deficiency in the A1-like phenotype by preventing Drp1-mediated excessive mitochondrial fission and subsequent mitochondrial malfunctions. Considering the neurotoxic roles of A1 astrocytes in primary neurons, we also confirm the effects of kir6.2-deficient A1 astrocytes on neuronal survival; the findings suggest that kir6.2^−/−^ A1 astrocytes exhibit impaired capability for inducing the damage of dopaminergic neurons. Therefore, the present study demonstrates a regulatory role for kir6.2 in driving excessive astrocyte activation in PD (Figure 9).

Neuroinflammation, as collective effects contributed by all glial cells, causes persistently detrimental damage to neuronal cells in chronic neurological diseases, especially degenerative disease including PD (Liddelow et al., 2020). The injurious factors of PD provoke robust and profound responses from glia via an inflammatory responses referred to as reactivity or activation. During this process, the microglia act as the initiators and astrocytes as the amplifiers (Kam et al., 2020; Liddelow et al., 2020). Although the molecular mechanisms underlying the action of glial cells in inflammatory responses in PD have been widely investigated, promising therapeutic targets for inflammatory regulation have yet to be discovered.

Previous studies have shown that kir6.2 is involved in regulating the electrical activity of dopaminergic neurons and dopamine release (Shi et al., 2008; Schiemann et al., 2012). This function may underlie the decreased exploratory behaviors and impaired locomotor activities of kir6.2^−/−^ mice (Deacon et al., 2006; Shi et al., 2008; Schiemann et al., 2012). These findings confirm the crucial roles of kir6.2-containing K-ATP channels in the dopamine system. Further studies have illustrated the theoretical foundation of kir6.2 as a target for PD, in which kir6.2 deficiency improved the pathological phenotypes of chronic MPTP mouse model for PD (Liss et al., 2005; Zhou et al., 2018). Owing to the sensitivity of kir6.2-containing KATP to metabolic stress, the mitochondria-targeting neurotoxin induced the continuous openning of neuronal kir6.2-KATP channels. This continuous opening results in the energy-saving inhibition of neuronal electrical activity and promotes the death of dopaminergic neurons in the long run (Dragicevic et al., 2015; Duda et al., 2016). We have previously shown that kir6.2 deletion alleviates neuronal iron metabolism and promotes the differentiation of neural precursors to neurons to protect mice from neurodegeneration (Zhang et al., 2018b; Zhou et al., 2018). These findings clarifies the causal link between kir6.2 and neurotoxin-induced PD pathology from different perspectives. The current study demonstrated that kir6.2 was also a critical mediator in the reactivity of mesencephalic astrocytes induced by inflammatory stimuli, which provided additional evidence of its significant role in PD progression. The current report indicated that as an aberrant phenomenon under LPS stimulation, the astrocytic kir6.2 led to an A1 neurotoxic astrocytic phenotype in PD. It is commonly recognized that the pore-forming subunits of KATP channels (kir6.1 and kir6.2) show specific distributions in different cell types of the brain, with kir6.2 mainly in neurons and kir6.1 in astrocytes (Thomzig et al., 2003). However, recent data indicated that substantial increases of kir6.2 expression in reactive astrocytes were identified in diseases including severe traumatic brain injury, brain contusions and 3×Tg-AD model for AD (Griffith et al., 2016; Castro et al., 2019; Gerzanich et al., 2019). Former studies have verified that KCNJ11, the encoding gene of kir6.2 protein, is regulated by NF-κB signaling in hepatocellular carcinoma and p38 MAPK/PKC signal pathways in AD (Zhang et al., 2018a; Li et al., 2019). We found in our research that LPS-activated microglia induced astrocytic kir6.2 expression, therefore we conjectured that inflammatory nuclear transcription factor including NF-κB may underlie the upregulation of astrocytic kir6.2. Nevertheless, the role of inducibly expressed kir6.2 in astrocytes of these pathological conditions remains unknown. Our current set of data corroborate that the expression of kir6.2 in astrocytes under LPS-induced PD pathological conditions is involved in the formation of neurotoxic astrocyte phenotype, which promotes a better understanding of kir6.2’s roles in astrocytic pathology.

Astrocytes, formerly recognized as passive supporters of neuronal functions, are actually active parts in the physiology and pathology of neurons. As the most abundant cell type in the central nervous system, astrocytes closely communicate with neurons through numerous processes. Thus, astrocytes participate in multi-faceted neuronal functions including metabolic homeostasis control, synapses pruning by phagocytosis, gliotransmission-mediated signal transduction, initiation and propagation of neuroinflammation, among others (Souza et al., 2019; Giovannoni and Quintana, 2020). Our former studies had an advantage in focusing on the roles of astrocytes in PD, including neurotransmitter regulation, neurotrophins synthesis, inflammasome formation and cellular antioxidant systems (Li et al., 2012; Zhu et al., 2018; Wei et al., 2020). The A1 astrocytic phenotype is a newly identified astrocyte subpopulation triggered by the LPS-activated microglia. This type of reactive astrocytes, abundant in normal aging and various neurodegenerative diseases, have lost most normal astrocytic functions but gain a new neurotoxic function by rapidly killing neurons (Clarke et al., 2018). A study has determined that pathological microglia-to-astrocyte-to-neuron mitochondrial dysfunction underlies the inflammation-induced death of neurons. During this process, A1 astrocytes are seriouly injured in mitochondrial functions but act as accurate transmitters of damage signal (Joshi et al., 2019). Astrocytic mitochondria exhibit active activity, not only for their own metabolism, but also providing energy-generation precursors to meet neuronal action potential requirements (McAvoy and Kawamata, 2019). Additionally, neurons depend on the supply of endogenous antioxidant systems from astrocytes for redox stress reduction; during this process, healthy astrocytic mitochondria function significantly (Baxter and Hardingham, 2016). In the current study, we observed the mitochondrial impairements of A1 astrocytes, as well as their neurotoxic roles after LPS-MCM treatment, which is consistent with former study. By recovering the mitochondrial malfunctions of astrocytes, kir6.2 deletion prevented the activation of A1 astrocytes, breaking the propagation of injury from astrocytes to neurons. Therefore, kir6.2 performed additional roles in regulating astrocytic functions, in addition to being generally regarded as a neuronal receptor, which would be a pleiotropic target for the pathology of PD.

Mitochondrial dynamics, including fission, fusion, transport and mitophagy render mitochondria highly mobile and functional. Mediated by Drp1, mitochondrial fission should be strictly controlled because excessive mitochondrial fission and fragmentation often occur in neurodegenerative diseases (Knott et al., 2008; Yan et al., 2020). Drp1-Fis1 fragmentation contributes to A1 astrocyte reactivity in mouse models for neurodegenerative diseases. Suppression of this process prevents neuronal degeneration (Joshi et al., 2019). As we found the inhibitory effects of kir6.2 deletion on excessive Drp1-dependent mitochondrial fission of A1 astrocytes, we suggested that kir6.2 deficiency act as a Drp1 inhibitor to interrupt mitochondrial impairments. Drp1-mediated mitochondrial fission is a process of mitochondrial localization of Drp1 from cytoplasm (Cho et al., 2013). Thus, we separated the cytoplasmic and mitochondrial components and detected the Drp1 levels in each part. The results showed that kir6.2 deficiency reduced the mitochondrial anchorage of Drp1, which at least in part revealed molecular mechanisms of kir6.2 in regulating mitochondrial functions of astrocytes. With regard to the reduction of Drp1-dependent mitochondrial fission when kir6.2 is absent, two conjectures are presented. First, as mitochondrial membranes express the functional KATP channel (Raval et al., 2007; Hawrysh et al., 2016; Paggio et al., 2019), the ectopic expression of kir6.2 in astrocytes may be a component of mitochondrial KATP which regulates organelle functions. Second, KATP in cell membranes regulates the cellular calcium signal that is crucial for mitochondrial quality control, including mitochondrial fission/fusion (Lovy et al., 2020; Zhang et al., 2020). Whether the underlying mechanisms are related to mitochondrial or cyto-membranous KATP needs to be further explored in future studies.

In summary, this study strongly proves the regulatory role of kir6.2 in the neuroinflammatory pathogenesis of PD. We reveal for the first time that inflammtory stimuli induce ectopic expression of kir6.2 in astrocytes, which is indespensible in the formation of the A1 astrocytes phenotype. Kir6.2 deficiency cancels neurotoxic astrocyte reactivity by recovering Drp1-dependent excessive mitochondrial fission, providing protection against neurodegeneration in PD. This study suggests that kir6.2 is a potential target for the development of a therapeutic approach to protecting dopaminergic neurons from astrocytic inflammation.

## Data Availability Statement

The authors acknowledge that the data presented in this study must be deposited and made publicly available in an acceptable repository, prior to publication. Frontiers cannot accept a article that does not adhere to our open data policies.

## Ethics Statement

The animal study was reviewed and approved by Review committee from Nanjing Medical University.

## Author Contributions

ML designed the research; NS, HZ, RX, JL, and JZ performed the experiment; YF reviewed and checked the experiment; JD provided technical support; NS analyzed the data and wrote the paper; ML revised the paper; GH edited the paper.

## Funding

This work was supported by the grants from the National Natural Science Foundation of China (No. 81922066, No. 81773706, No. 81991523 and No. 81630099) and the Drug Innovation Major Project (No. 2018ZX09711001-003-007).

## Conflict of Interest

The authors declare that the research was conducted in the absence of any commercial or financial relationships that could be construed as a potential conflict of interest.

## References

[B1] Batista C. Gomes G. F. Candelario-Jalil E. Fiebich B. L. de Oliveira A. (2019). Lipopolysaccharide-induced neuroinflammation as a bridge to understand neurodegeneration. Int. J. Mol. Sci. 20. 10.3390/ijms20092293 PMC6539529 31075861

[B2] Baxter P. S. Hardingham G. E. (2016). Adaptive regulation of the brain’s antioxidant defences by neurons and astrocytes. Free Radic. Biol. Med. 100, 147–152. 10.1016/j.freeradbiomed.2016.06.027 27365123 PMC5145800

[B3] Bernaus A. Blanco S. Sevilla A. (2020). Glia crosstalk in neuroinflammatory diseases. Front. Cell. Neurosci. 14, 209. 10.3389/fncel.2020.00209 32848613 PMC7403442

[B4] Castro L. Noelia M. Vidal-Jorge M. Sánchez-Ortiz D. Gándara D. Martínez-Saez E. (2019). Kir6.2, the pore-forming subunit of ATP-sensitive K+ channels, is overexpressed in human posttraumatic brain contusions. J. Neurotraum. 36, 165–175. 10.1089/neu.2017.5619 PMC7872003 29737232

[B5] Cho B. Choi S. Y. Cho H. M. Kim H. J. Sun W. (2013). Physiological and pathological significance of dynamin-related protein 1 (Drp1)-Dependent mitochondrial fission in the nervous system. Experimental Neurobiology 22, 149–157. 10.5607/en.2013.22.3.149 24167410 PMC3807002

[B6] Clarke L. E. Liddelow S. A. Chakraborty C. Münch A. E. Heiman M. Barres B. A. (2018). Normal aging induces A1-like astrocyte reactivity. Proc. Natl. Acad. Sci. Unit. States Am. 115, E1896–E1905. 10.1073/pnas.1800165115 PMC5828643 29437957

[B7] Cunnane S. C. Trushina E. Morland C. Prigione A. Casadesus G. Andrews Z. B. (2020). Brain energy rescue: an emerging therapeutic concept for neurodegenerative disorders of ageing. Nat. Rev. Drug Discov. 19, 609–633. 10.1038/s41573-020-0072-x 32709961 PMC7948516

[B8] Deacon R. M. J. Brook R. C. Meyer D. Haeckel O. Ashcroft F. M. Miki T. (2006). Behavioral phenotyping of mice lacking the KATP channel subunit Kir6.2. Physiol. Behav. 87, 723–733. 10.1016/j.physbeh.2006.01.013 16530794

[B9] Domingues A. V. Pereira I. M. Vilaça-Faria H. Salgado A. J. Rodrigues A. J. Teixeira F. G. (2020). Glial cells in Parkinson´s disease: protective or deleterious? Cell. Mol. Life Sci. 77, 5171–5178. 10.1007/s00018-020-03584-x 32617639 PMC11104819

[B10] Dragicevic E. Schiemann J. Liss B. (2015). Dopamine midbrain neurons in health and Parkinson’s disease: emerging roles of voltage-gated calcium channels and ATP-sensitive potassium channels. Neuroscience 284, 798–814. 10.1016/j.neuroscience.2014.10.037 25450964

[B11] Du R. Tan J. Yan N. Wang L. Qiao C. Ding J. (2014). Kir6.2 knockout aggravates lipopolysaccharide-induced mouse liver injury via enhancing NLRP3 inflammasome activation. J. Gastroenterol. 49, 727–736. 10.1007/s00535-013-0823-0 23771404

[B12] Duda J. Pötschke C. Liss B. (2016). Converging roles of ion channels, calcium, metabolic stress, and activity pattern ofSubstantia nigra dopaminergic neurons in health and Parkinson's disease. J. Neurochem. 139, 156–178. 10.1111/jnc.13572 26865375 PMC5095868

[B13] Duty S. Jenner P. (2011). Animal models of Parkinson's disease: a source of novel treatments and clues to the cause of the disease. Br. J. Pharmacol. 164, 1357–1391. 10.1111/j.1476-5381.2011.01426.x 21486284 PMC3229766

[B14] Gerzanich V. Stokum J. A. Ivanova S. Woo S. K. Tsymbalyuk O. Sharma A. (2019). Sulfonylurea receptor 1, transient receptor potential cation channel subfamily M member 4, and KIR6.2:role in hemorrhagic progression of contusion. J. Neurotraum. 36, 1060–1079. 10.1089/neu.2018.5986 PMC6446209 30160201

[B15] Giovannoni F. Quintana F. J. (2020). The role of astrocytes in CNS inflammation. Trends Immunol *.* 41, 805–819. 10.1016/j.it.2020.07.007 32800705 PMC8284746

[B16] Griffith C. M. Xie M. Qiu W. Sharp A. A. Ma C. Pan A. (2016). Aberrant expression of the pore-forming KATP channel subunit Kir6.2 in hippocampal reactive astrocytes in the 3xTg-AD mouse model and human Alzheimer's disease. Neuroscience 336, 81–101. 10.1016/j.neuroscience.2016.08.034 27586053

[B17] Hawrysh P. J. Miles A. R. Buck L. T. (2016). Phosphorylation of the mitochondrial ATP-sensitive potassium channel occurs independently of PKCε in turtle brain. Comp. Biochem. Physiol. B Biochem. Mol. Biol. 200, 44–53. 10.1016/j.cbpb.2016.06.002 27280321

[B18] Hinkle J. T. Dawson V. L. Dawson T. M. (2019). The A1 astrocyte paradigm: new avenues for pharmacological intervention in neurodegeneration. Mov. Disord. 34, 959–969. 10.1002/mds.27718 31136698 PMC6642014

[B19] Jha M. K. Kim J. H. Song G. J. Lee W. H. Lee I. K. Lee H. W. (2018). Functional dissection of astrocyte-secreted proteins: implications in brain health and diseases. Prog. Neurobiol. 162, 37–69. 10.1016/j.pneurobio.2017.12.003 29247683

[B20] Joshi A. U. Minhas P. S. Liddelow S. A. Haileselassie B. Andreasson K. I. Dorn G. W. (2019). Fragmented mitochondria released from microglia trigger A1 astrocytic response and propagate inflammatory neurodegeneration. Nat. Neurosci. 22, 1635–1648. 10.1038/s41593-019-0486-0 31551592 PMC6764589

[B21] Kam T. Hinkle J. T. Dawson T. M. Dawson V. L. (2020). Microglia and astrocyte dysfunction in Parkinson's disease. Neurobiol. Dis. 144, 105028. 10.1016/j.nbd.2020.105028 32736085 PMC7484088

[B22] Knott A. B. Perkins G. Schwarzenbacher R. Bossy-Wetzel E. (2008). Mitochondrial fragmentation in neurodegeneration. Nat. Rev. Neurosci. 9, 505–518. 10.1038/nrn2417 18568013 PMC2711514

[B23] Li Y. Ba M. Du Y. Xia C. Tan S. Ng K. P. (2019). Aβ1-42 increases the expression of neural KATP subunits Kir6.2/SUR1 via the NF-κB, p38 MAPK and PKC signal pathways in rat primary cholinergic neurons. Hum. Exp. Toxicol. 38, 665–674. 10.1177/0960327119833742 30868916

[B24] Li Y. Wang F. Wang W. Luo Y. Wu P. Xiao J. (2012). Aquaporin-4 deficiency impairs synaptic plasticity and associative fear memory in the lateral amygdala: involvement of downregulation of glutamate transporter-1 expression. Neuropsychopharmacology (New York, N.Y.) 37, 1867–1878. 10.1038/npp.2012.34 PMC3376319 22473056

[B25] Liddelow S. A. Guttenplan K. A. Clarke L. E. Bennett F. C. Bohlen C. J. Schirmer L. (2017). Neurotoxic reactive astrocytes are induced by activated microglia. Nature 541, 481–487. 10.1038/nature21029 28099414 PMC5404890

[B26] Liddelow S. A. Marsh S. E. Stevens B. (2020). Microglia and astrocytes in disease: dynamic duo or partners in crime? Trends Immunol *.* 41, 820–835. 10.1016/j.it.2020.07.006 32819809

[B27] Liss B. Haeckel O. Wildmann J. Miki T. Seino S. Roeper J. (2005). K-ATP channels promote the differential degeneration of dopaminergic midbrain neurons. Nat. Neurosci. 8, 1742–1751. 10.1038/nn1570 16299504

[B28] Lovy A. Ahumada-Castro U. Bustos G. Farias P. Gonzalez-Billault C. Molgo J. (2020). Concerted action of AMPK and sirtuin-1 induces mitochondrial fragmentation upon inhibition of Ca(2+) transfer to mitochondria. Front Cell. Dev. Biol. 8, 378. 10.3389/fcell.2020.00378 32523953 PMC7261923

[B29] McAvoy K. Kawamata H. (2019). Glial mitochondrial function and dysfunction in health and neurodegeneration. Mol. Cell. Neurosci. 101, 103417. 10.1016/j.mcn.2019.103417 31678567

[B30] Paggio A. Checchetto V. Campo A. Menabò R. Di Marco G. Di Lisa F. (2019). Identification of an ATP-sensitive potassium channel in mitochondria. Nature 572, 609–613. 10.1038/s41586-019-1498-3 31435016 PMC6726485

[B31] Poewe W. Seppi K. Tanner C. M. Halliday G. M. Brundin P. Volkmann J. (2017). Parkinson disease. Nat. Rev. Dis. Primers. 3, 17013. 10.1038/nrdp.2017.13 28332488

[B32] Raval A. P. Dave K. R. DeFazio R. A. Perez-Pinzon M. A. (2007). ɛPKC phosphorylates the mitochondrial K+ATP channel during induction of ischemic preconditioning in the rat hippocampus. Brain Res *.* 1184, 345–353. 10.1016/j.brainres.2007.09.073 17988655 PMC2577914

[B33] Schiemann J. Schlaudraff F. Klose V. Bingmer M. Seino S. Magill P. J. (2012). K-ATP channels in dopamine substantia nigra neurons control bursting and novelty-induced exploration. Nat. Neurosci. 15, 1272–1280. 10.1038/nn.3185 22902720 PMC4242970

[B34] Shi X. Chang J. Ding J. Fan Y. Sun X. Hu G. (2008). Kir6.2 knockout alters neurotransmitter release in mouse striatum: an *in vivo* microdialysis study. Neurosci. Lett. 439, 230–234. 10.1016/j.neulet.2008.05.024 18524485

[B35] Souza D. G. Almeida R. F. Souza D. O. Zimmer E. R. (2019). The astrocyte biochemistry. Semin. Cell Dev. Biol. 95, 142–150. 10.1016/j.semcdb.2019.04.002 30951895

[B36] Thomzig A. Prüss H. Veh R. W. (2003). The Kir6.1-protein, a pore-forming subunit of ATP-sensitive potassium channels, is prominently expressed by giant cholinergic interneurons in the striatum of the rat brain. Brain Res *.* 986, 132–138. 10.1016/S0006-8993(03)03222-0 12965237

[B37] Tinker A. Aziz Q. Li Y. Specterman M. (2018). ATP-sensitive potassium channels and their physiological and pathophysiological roles. Comp. Physiol. 8, 1463–1511. 10.1002/cphy.c170048 30215858

[B38] Wei Y. Lu M. Mei M. Wang H. Han Z. Chen M. (2020). Pyridoxine induces glutathione synthesis via PKM2-mediated Nrf2 transactivation and confers neuroprotection. Nat. Commun. 11, 941–1012. 10.1038/s41467-020-14788-x 32071304 PMC7029000

[B39] Yan X. Wang B. Hu Y. Wang S. Zhang X. (2020). Abnormal mitochondrial quality control in neurodegenerative diseases. Front. Cell. Neurosci. 14, 138. 10.3389/fncel.2020.00138 32655368 PMC7324542

[B40] Yang Q. Q. Zhou J. W. (2019). Neuroinflammation in the central nervous system: symphony of glial cells. Glia 67, 1017–1035. 10.1002/glia.23571 30548343

[B41] Yun S. P. Kam T. Panicker N. Kim S. Oh Y. Park J. (2018). Block of A1 astrocyte conversion by microglia is neuroprotective in models of Parkinson's disease. Nat. Med. 24, 931–938. 10.1038/s41591-018-0051-5 29892066 PMC6039259

[B42] Zhang K. Mu L. Ding M. Xu R. Ding Z. Liang J. (2018a). NFκB mediated elevation of KCNJ11 promotes tumor progression of hepatocellular carcinoma through interaction of lactate dehydrogenase A. Biochem. Bioph. Res. Co. 495, 246–253. 10.1016/j.bbrc.2017.11.011 29108994

[B43] Zhang Q. Li C. Zhang T. Ge Y. Han X. Sun S. (2018b). Deletion of Kir6.2/SUR1 potassium channels rescues diminishing of DA neurons via decreasing iron accumulation in PD. Mol. Cell. Neurosci. 92, 164–176. 10.1016/j.mcn.2018.08.006 30171894

[B44] Zhang X. Griepentrog J. E. Zou B. Xu L. Cyr A. R. Chambers L. M. (2020). CaMKIV regulates mitochondrial dynamics during sepsis. Cell Calcium 92, 102286. 10.1016/j.ceca.2020.102286 32932146 PMC7686261

[B45] Zhou Y. Zhu J. Lv Y. Song C. Ding J. Xiao M. (2018). Kir6.2 deficiency promotes mesencephalic neural precursor cell differentiation via regulating miR-133b/GDNF in a Parkinson’s disease mouse model. Mol. Neurobiol. 55, 8550–8562. 10.1007/s12035-018-1005-0 29564810

[B46] Zhu J. Hu Z. Han X. Wang D. Jiang Q. Ding J. (2018). Dopamine D2 receptor restricts astrocytic NLRP3 inflammasome activation via enhancing the interaction of β-arrestin2 and NLRP3. Cell Death Differ. 25, 2037–2049. 10.1038/s41418-018-0127-2 29786071 PMC6219479

