# Corrigendum: Induced expression of kir6.2 in A1 astrocytes propagates inflammatory neurodegeneration *via* Drp1-dependent mitochondrial fission

**DOI:** 10.3389/fphar.2023.1111925

**Published:** 2023-03-29

**Authors:** Nanshan Song, Hong Zhu, Rong Xu, Jiaqi Liu, Yinquan Fang, Jing Zhang, Jianhua Ding, Gang Hu, Ming Lu

**Affiliations:** ^1^ Jiangsu Key Laboratory of Neurodegeneration, Department of Pharmacology, Nanjing Medical University, Nanjing, China; ^2^ Department of Pharmacology, Nanjing University of Chinese Medicine, Nanjing, China; ^3^ Neuroprotective Drug Discovery Key Laboratory, Department of Pharmacology, Nanjing Medical University, Nanjing, China

**Keywords:** neuroinflammation, astrocytes, mitochondrial fission, Parkinson’s diseases, kir6.2

In the published article, there was an error in Figure 6 as published. Due to the unsuccessful replacement of the Western blotting band based on **Figure 4H** as the lay-out template, the β-actin band in Figure 6C was inadvertently duplicated with the β-actin band in **Figure 4H**. Similarly, the original legend of Figure 6C was a repeated writing of the legend in **Figure 4H** and displayed as “(C) Expression of C3 in the midbrain were detected by Western blotting and its densitometric analysis.” The correct legend is “(C) Expression of C3 in primary astrocytes detected by Western blotting and its densitometric analysis.” From a more rigorous perspective, we reviewed the statistical data and re-plotted the statistical histogram in Figure 6C. The corrected Figure 6C and the legend appear below. The authors provided the journal with the source data files. Results and conclusions were not affected.

**FIGURE 6:**
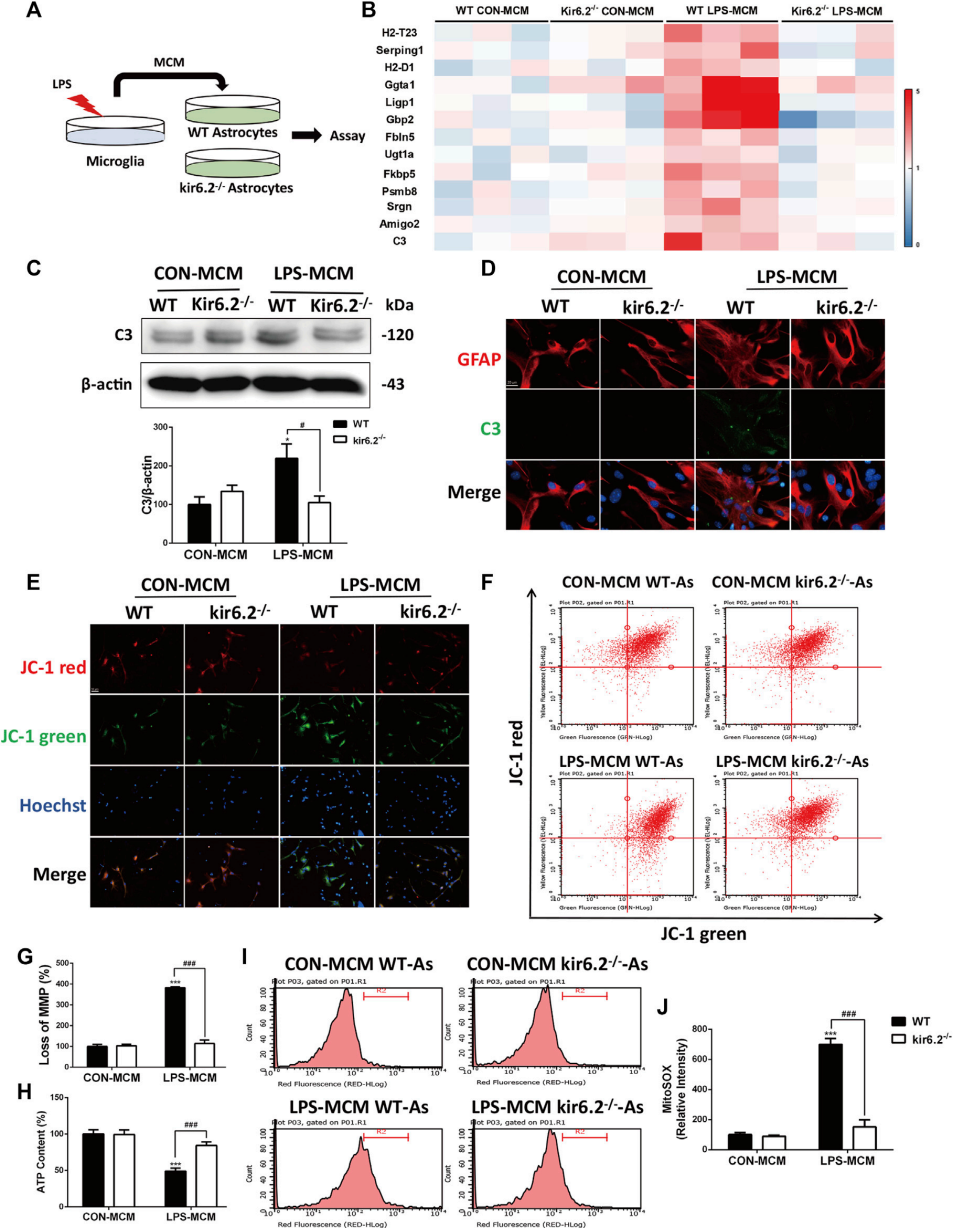
Kir6.2-deficient astrocytes are resistant to neurotoxic A1-like phenotype *in vitro*. **(A)** Protocol of treatment for **(B–J)**. Primary microglia from WT mice were stimulated with 100 ng/mL LPS for 24 h to collect the MCM. For primary astrocytes cultures, the MCM was diluted at a ratio of 1:3 to incubate the primary astrocytes from WT and kir6.2^−/−^ mice for 24 h. **(B)** Heat map comparing the mean expression of A1-specific transcripts in astrocytic RNA samples by RT-PCR. **(C)** Expression of C3 in primary astrocytes detected by Western blotting and its densitometric analysis. **(D)** Immunofluorescent stainings of C3 (green) and GFAP (red) in primary astrocytes. **(E)** Representative images of JC-1 stain in astrocytes were observed by confocal microscopy. Hoechst stains nucleus (blue). **(F)** Flow cytometric analysis of astrocytes stained with JC-1 fluorescent probe. **(G)** Quantification of MMP loss in JC-1 staining measured by flow cytometry. **(H)** ATP contents of astrocytes were analyzed. **(I)** Astrocytes were stained with MitoSOX fluorescent probe and analyzed by flow cytometry. **(J)** Quantification of the mitochondrial ROS by MitoSOX staining. Data were analyzed using two-way ANOVA. *p < 0.05 and ***p < 0.001 vs. corresponding CON-MCM group. ^#^ *p* < 0.05 and ^###^ *p* < 0.001 vs. WT LPS-MCM group. Values are presented as means ± SEM from three independent experiments.

The authors apologize for this error and state that this does not change the scientific conclusions of the article in any way. The original article has been updated.

